# Phyto-Mediated Zinc Oxide Nanoparticles from *Raphanus sativus* (L.): Metabolomic Insights, Gastroprotective Potential, and Docking-Supported Evidence

**DOI:** 10.3390/life15111710

**Published:** 2025-11-05

**Authors:** Doaa K. Alsayed, Seham S. El-Hawary, Mohamed A. El Raey, Gihan Fouad, Mohamed F. Abdelhameed, Ahmed F. Essa, Yasmine H. Ahmed, Saad A. Alshehri, Mohamed A. Rabeh, Amira K. Elmotayam

**Affiliations:** 1 Department of Pharmacognosy, National Nutritional Institute, General Organization for Teaching Hospitals and Institutes, Kasr El-Aini, Cairo 12613, Egypt; 2 Department of Pharmacognosy, Faculty of Pharmacy, Cairo University, Kasr El-Aini, Cairo 11562, Egypt; seham.elhawary@yahoo.com; 3 Phytochemistry and Plant Systematics Department, Pharmaceutical and Therapeutical Industries Research Institute, National Research Centre, Dokki, Cairo 12311, Egypt; elraiy@gmail.com; 4 Department of Clinical Nutrition, National Nutritional Institute, General Organization for Teaching Hospitals and Institutes, Kasr El-Aini, Cairo 12613, Egypt; gihan_fouad@yahoo.com; 5 Pharmacology Department, Medical Research and Clinical Studies Institute, National Research Centre, Dokki, Cairo 12311, Egypt; fayed.nrc@gmail.com; 6 Chemistry of Natural Compounds Department, National Research Centre, Dokki, Giza 12622, Egypt; ahmedfathyessa551@gmail.com; 7 Cytology and Histology Department, Veterinary Medicine Faculty, Cairo University, Cairo 11562, Egypt; yasmine_hamdi@cu.edu.eg; 8 Department of Pharmacognosy, College of Pharmacy, King Khalid University, Abha 62251, Saudi Arabia; salshhri@kku.edu.sa (S.A.A.); mrabeh@kku.edu.sa (M.A.R.)

**Keywords:** gastritis, green synthesis, phytochemicals, plant extract, zinc oxide nanoparticles, oxidative stress, inflammation

## Abstract

This study aimed to synthesize zinc oxide nanoparticles (ZnO-NPs) via a green sustainable approach using *Raphanus sativus* (L.) root extract and evaluate their gastroprotective effect against ethanol-induced gastric injury in rats. ZnO-NPs were characterized through UV–Vis spectroscopy, FT-IR, TEM, zeta potential analysis, and XRD. LC- MS-coupled metabolic profiling was employed to identify different phytochemical compounds in the plant. Oxidative stress biomarkers (GSSG, GPX, and CAT), gastric secretions (gastrin and histamine), inflammatory cytokines (TNF-α and NF-κB), and molecular markers (MMP-10 and pERK1/2) were evaluated. Treatment with ZnO-NPs and plant extract restored antioxidant enzyme activity in a dose-dependent manner and decreased oxidative and inflammatory markers. Histopathological and histochemical analyses confirmed the protection of the gastric mucosa. The ZnO-NPs at (200 mg/kg), showed superior efficacy over the extract and, in some cases, displayed equivalent or enhanced effects relative to the reference drug omeprazole. In silico findings support the gastroprotective potential of the plant by demonstrating strong binding associations for major phytochemicals. This paper highlights that green-synthesized ZnO-NPs exhibit a significant gastroprotective effect through the modulation of oxidative stress and inflammatory pathways, indicating their promise as a safe and effective alternative treatment for gastric ulcers.

## 1. Introduction

Gastritis, a widespread disease of the gastrointestinal tract, is distinguished by inflammation and erosion of the stomach mucosa, often associated with various digestive consequences such as ulcers, bleeding, and gastric tumors. It is broadly classified into acute and chronic gastritis. The acute form develops suddenly and is characteristically short-lived. It is often caused by numerous factors, including infections, trauma, ischemia, and medications [1]. In contrast, chronic gastritis develops gradually and persists over a prolonged period. Approximately half of the worldwide population suffers from chronic gastritis, with a strong association to *Helicobacter pylori* infection [2]. Recent therapeutic approaches for gastritis involve monotherapy or combination therapy using antibiotics, proton pump inhibitors (PPIs), H_2_ receptor blockers, and prostaglandin analogs. These treatments are associated with considerable adverse effects, which reduce their long-term use. Thus, there is a growing demand to explore novel therapeutic strategies that offer greater efficacy and safety. Natural products derived from plants exhibit low toxicity, great bioavailability, and negligible drug resistance.

*Raphanus sativus* (L.), often called radish [3], is classified under the Brassicaceae family. It is a root vegetable broadly consumed in salads with great nutritional benefits due to its crunchy texture and refreshing taste. Numerous cultivars differ in size, color, and the time required to mature [4]. The outer surface of the root shows a varied range of colors, including white, purple, and red, which contribute to its phytochemical composition. Several reports have identified bioactive secondary metabolites in this plant, including phenolic compounds, glucosinolates, and flavonoids [5]. *R. sativus* has notable anti-inflammatory and gastroprotective properties that may help in reducing gastric inflammation, suppressing free radical formation, and promoting gastric mucosal repair [6].

Currently, plants represent the best source for the large-scale green synthesis of nanoparticles as a result of their rapid production with diverse morphologies and sizes. Zinc oxide is an excellent material for producing nanostructures with unique biological activities and structural arrangements [7]. Zinc oxide nanoparticles (ZnO-NPs) have numerous advantages, including being cost-effective, efficiently absorbed by biological tissue, and time-saving [8]. Also, the FDA (US Food and Drug Administration) classified it as “GRAS” (generally regarded as safe) [9]. Additionally, ZnO-NPs reveal promising and broad-spectrum biomedical applications, including anti-inflammatory, anticancer, antibacterial, and anti-diabetic properties [10]. ZnO-NPs are considered an efficient drug delivery platform for treating numerous diseases due to their ability to deliver drugs efficiently to different cellular targets [11].

Our paper is designed to synthesize ZnO-NPs utilizing the root extract of *Raphanus sativus* (L.) through an eco-friendly, green chemistry-based approach. The produced nanoparticles are characterized through UV analysis, X-ray diffraction (XRD), zeta potential (ZP), transmission electron microscopy (TEM), and Fourier-transform infrared spectroscopy (FT-IR) to confirm their morphological and structural properties. In addition, the gastroprotective role of the extract and the formulation of ZnO-NPs was evaluated in rats against ethanol-induced gastritis. The phytochemical profiling of *R. sativus* root extract is accomplished to identify the active constituents using ultra-performance liquid chromatography coupled with quadrupole time-of-flight tandem mass spectrometry (UPLC-QTOF-MS/MS). Docking simulations were applied to support the observed gastroprotective effect through the binding affinities of the identified compounds with the active sites of key proteins and provide mechanistic insights for their activity. Therefore, this study determines a sustainable approach for nanoparticle synthesis and provides appreciated insights into the possible role of ZnO-NPs as a novel gastroprotective agent. Specifically, our work aimed to evaluate the gastroprotective mechanisms of green-synthesized ZnO-NPs against ethanol-induced gastric injury, supported by metabolomic and molecular docking analyses. The findings of this work are expected to contribute to the improvement of innovative, biocompatible therapeutic approaches in nanomedicine and gastrointestinal health research.

## 2. Materials and Methods

### 2.1. Plant Material Collection and Ethical Compliance

In December 2024, *R. sativus* roots were collected from the Agricultural Research Center, Cairo, Egypt. A voucher specimen (2-12-2024-F) was archived in the herbarium of the Pharmacognosy Department, Faculty of Pharmacy, Cairo University, Giza, Egypt. All experimental procedures were approved by the Research Ethics Committee for Experimental and Clinical Studies, Faculty of Pharmacy, Cairo University, Egypt (approval code: MP (3741); approval date: 25 November 2024).

### 2.2. Preparation of the Plant Extract

Roots of *R. sativus* were homogenized with ethanol using a blender at a ratio of 100 g/L of ethanol, repeated three times. The extract was filtered and concentrated under vacuum at 40 °C to obtain a sticky residue, then preserved in an opaque container at 4 °C for subsequent analysis.

### 2.3. Metabolic Profiling and Molecular Network

Identification the metabolic constituents of the root extract of *R. sativus* was conducted using LC-ESI-TOF-MS [12]. Global Natural Product Social (GNPS) was used to study LC-MS-MS data of the plant extract using the molecular networking approach [13].

#### 2.3.1. Sample Preparation

A quantity of 50 mg of the extract was mixed in 1 mL of reconstitution solvent, which comprised water, methanol, and acetonitrile in a ratio of (H_2_O:MeOH:ACN 50:25:25, *v*/*v*/*v*) to prepare a stock solution. Then, the solution was homogenized and underwent ultrasonication at 30 kHz for 10 min, followed by centrifugation for 10 min at 10,000 rpm. Afterward, 50 µL of the supernatant was adjusted to 1000 µL, yielding an ultimate injected concentration of 2.5 µg/µL. Also, this procedure was applied to both the quality control (QC) and blank, including an internal standard (IS). Sample injection (10 µL) was conducted in negative ionization mode.

#### 2.3.2. Instrumentation and Analytical Conditions

An Exion liquid chromatography (LC) instrument (AB Sciex, Framingham, MA, USA) coupled to an autosampler was employed for chromatographic separation. The pre-column in-line filter was utilized (0.5 µm × 3.0 mm, Phenomenex, Torrance, CA, USA) and an Xbridge C18 was used (3.5 µm, 2.1 × 50 mm, Waters Corporation, Milford, MA, USA). The temperature was set to 40 °C and the flow rate was controlled at 300 µL/min. The mobile phase was composed of two solutions. Solution A consisted of 5 mM ammonium formate in 1% methanol, adjusted to pH 3.0 with formic acid, whereas Solution B contained 5 mM ammonium formate in 1% methanol, adjusted to pH 8.0 with sodium hydroxide. The gradient elution was applied according to the following program: 0–20 min: 10% B, 21–25 min: 90% B, 25.01–28 min: 10% B, and 28 min onward: 90% B (column equilibration). A mass spectrometric (MS) study was operated in negative electrospray ionization (ESI) mode. The MS parameters were set to ensure precise detection. The declustering potential was optimized at −80 V, while the sprayer capillary voltage was set at −4500 V. The curtain gas pressure was fixed at 25 psi, and the source temperature was maintained at 600 °C. Both gas 1 and gas 2 pressures were adjusted to 40 psi. A collision energy of −35 V with a spread of 20 V was controlled. Ion tolerance was regulated at 10 ppm with a high-resolution survey scan range of 50–1100 *m*/*z* and survey scan acquisition time of 50 ms.

#### 2.3.3. LC-MS Data Processing

Metabolite identification was performed using MS-DIAL 4.8 open-source software and was conducted by matching spectral data against the ReSpect negative mode database, which contains 1573 records. Peak recognition from the total ion chromatogram (TIC) was carried out using MasterView 1.1 software (AB SCIEX). Several measures were utilized to confirm metabolite identification, including a signal-to-noise proportion (>5) and a sample-to-blank relative intensity (>3).

### 2.4. Green Synthesis of Zinc Oxide Quantum Dots

Through an environmentally friendly approach, ZnO-NPs were successfully synthesized using the ethanolic extract of *R. sativus* roots as a natural reducing, capping, and stabilizing agent. One gram of the extract was dissolved in 100 mL of a hydroalcoholic solution and then mixed with 5 g of zinc acetate, which had been previously dissolved in bi-distilled water. The mixture was subjected to a controlled-temperature bath for 20 min at 100 °C. Ammonium hydroxide was used by gradual addition to adjust the pH to 12, which led to the precipitation of a white solid and confirmation of ZnO-NP synthesis. The reaction was adjusted at 100 °C for an additional 20 min to ensure complete formation. Centrifugation at 4000 rpm for 10 min was used to isolate the resulting precipitate. To remove any remaining impurities, the precipitate was rinsed twice with distilled water and absolute ethanol. Then, the product was lyophilized and stored at ambient temperature for additional characterization.

### 2.5. Characterization of Zinc Oxide Nanoparticles

#### 2.5.1. UV–Vis Spectral Analysis

A spectrophotometer (model UV-1601, Shimadzu Corporation, Kyoto, Japan) was employed to estimate ZnO-NPs. The range of wavelength used was between 200 and 800 nm.

#### 2.5.2. FT-IR Analysis

A Shimadzu FT-IR Affinity-1 Spectrometer (Shimadzu Corporation, Kyoto, Japan) was utilized to identify the characteristic groups involved in the synthesis and stabilization of ZnO-NPs. The samples were prepared as KBr pellets and scanned in the 4000–400 cm^−1^ range in triplicate.

#### 2.5.3. Zeta-Sizer Measurements

For determining the nanoscale size, a Zeta-sizer Nano-ZS laser diffractometer (Malvern, Worcestershire, UK) was manipulated. The nanoparticle suspension was prepared by dissolving 1 mg of ZnO-NPs in 10 mL of distilled water. An aliquot of 1 mL from this suspension was further diluted to a final volume of 3 mL with distilled water.

#### 2.5.4. Transmission Electron Microscopy (TEM) Analysis

The shape and particle size of the synthesized ZnO-NPs were investigated via utilizing transmission electron microscopy (TEM) (JEOL-JEM-1011, JEOL Ltd., Tokyo, Japan). Small drops of ZnO-NPs suspension were put on a carbon film-coated copper grid, then allowed to evaporate at room temperature before taking TEM images.

#### 2.5.5. X-Ray Diffraction (XRD)

X-ray diffraction (XRD) analysis was performed on the ZnO-NP powder using a Bruker D8 Advance diffractometer (Bruker AXS, Karlsruhe, Germany) to confirm the structural features and crystalline quality. The procedures were obtained across an extension of 2θ angles to identify characteristic peaks related to ZnO-NPs phases.

### 2.6. Bioassay

#### 2.6.1. Drugs and Chemicals

Omez^®^ is a formulation produced by Pharaonia Company, Alexandria, Egypt. The crucial chemicals used in our work included ethanol (purity > 99%; CAS# 64-17-5, Merck Millipore, Burlington , MA, USA), ketamine, and xylazine (both procured from BioVision Inc., Milpitas Boulevard, CA, USA). Catalase (CAT) enzyme (Catalog # K773-100; 100 reactions; procured from BioVision Inc., Milpitas, CA, USA), glutathione peroxidase (GPX) (Catalog # E1172Ra, procured from BT LAB, Shanghai, China), and oxidized glutathione (GSSG) (Catalog # EK721431, procured from AFG Bioscience, North York, ON, Canada) were used. Both gastrin (Catalog # E-El-R0472) and histamine (Catalog # E-El-0032) were obtained from (Elabscience Biotechnology Inc., Houston, TX, USA). BioLegend Inc., San Diego, CA, USA manufactured Tumor Necrosis Factor-Alpha (TNF-*α*) (Catalog # 438204; 5 plates), while Nuclear Factor Kappa (NF-κB) was purchased from (Elabscience Biotechnology Inc., Houston, TX, USA, Catalog # E-EL-R0674). Moreover, the levels of MMP-10 and pERK1/2 were evaluated using a polymerase chain reaction (PCR). (Thermo Fisher Scientific, Waltham, MA, USA) supplied the SuperScript IV One-Step RT-PCR Kit for the PCR.

#### 2.6.2. Experimental Animals

This study utilized thirty-five adult male Sprague Dawley rats, each body weight 150–200 g, taken from the National Research Centre, Dokki-Giza, Egypt. Prior to experimentation, the animals were acclimated under controlled conditions for seven days. Rats were kept in controlled conditions at a temperature of 24–27 °C and were exposed to a 12 h period of light and darkness. They were allowed to have an unlimited intake of water and diet during the study. Animal supervision and parameters were followed close to the ethical guidelines and approved protocols.

#### 2.6.3. Acute Toxicity Test

Male and female Swiss albino mice (aged two months) with an average body weight of 20.4 g were utilized for assessing the acute toxicity of *R. sativus* root extract and ZnO-NPs. The study followed previous research, which describes the procedure [14]. The mice were adapted for five days before experimentation and went through overnight fasting before administration of the drugs. An oral dose of 2000 mg/kg of the tested material was administered. To notice any abnormalities, signs were conducted on functional parameters including the eyes, ears, mucous membranes, skin, and the respiration and circulatory systems. Also, behavioral changes such as diarrhea, convulsions, salivation, and tremors were carefully observed. There were no deaths or observations of toxic response up to the highest administered dose (2000 mg/kg body weight). These findings suggest that the extract is regarded as safe for use.

#### 2.6.4. Experimental Design

**Figure 1:**
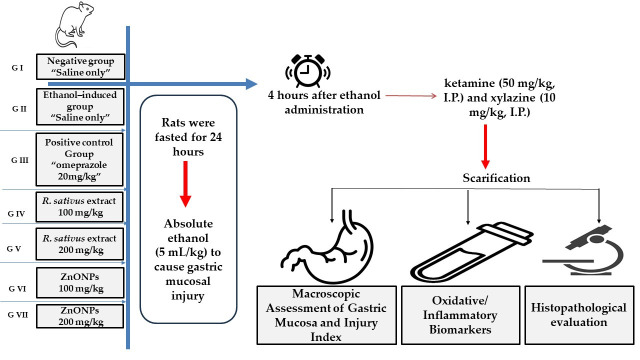
A schematic diagram of the experimental design.

The study included seven groups of rats (n = 5 per group). Group I represented the healthy control (negative group), consisting of rats with no induced gastritis. Group II acted as an untreated control, in which gastritis was induced, but no treatment was administered (ethanol-induced group). Group III functioned as the positive control, where ethanol-induced gastritis model rats were orally administered omeprazole (20 mg/kg). Groups IV and V comprised ethanol-induced gastritis model rats administered two dose levels of *R. sativus* root extract, 100 mg/kg and 200 mg/kg, respectively. Group VI included ethanol-induced gastritis rats administered ZnO-NPs at 100 mg/kg, while Group VII were given ZnO-NPs at 200 mg/kg. A schematic diagram for the experimental design is displayed in Figure 1.

#### 2.6.5. Gastritis Induction

Gastritis was induced in all relevant groups following the previously described protocol of [15]. Rats were fasted for 24 h to optimize experimental conditions, then absolute ethanol (5 mL/kg) was administered orally to cause gastric mucosal injury.

#### 2.6.6. Macroscopic Assessment of Gastric Mucosa and Injury Index

Animals were administered ketamine (50 mg/kg, I.P.) and xylazine (10 mg/kg, I.P.) four hours after ethanol administration. The animals were then sacrificed and their stomachs were carefully excised. The excised stomach tissues were immediately rinsed with chilled phosphate-buffered saline (PBS) to facilitate further evaluation of gastric damage and to assess the severity of ulcers. Ulcer severity (US) and ulcer number (UN) were used as standard parameters for inflammation and ulcer formation. The size and intensity of ulcers were analyzed based on photographic documentation of the excised stomachs. The ulceration grade was evaluated to measure the degree of gastric mucosal damage from 0 to 5, following the Guth guidelines. Where 0 (normal mucosa), 1 (vascular congestions; red coloration), 2 (one–two lesions; spot ulcer), 3 (severe injury; hemorrhagic streaks), 4 (very severe injury; deep ulcer), and 5 (mucosa full of lesion; perforated ulcer) [16]. The ulcer index (UI) was estimated via Formula [17]:

$$UI=(UN+US+UP)\times 10^{-1}$$

UN refers to the mean ulcer count per rat, US corresponds to the average severity rating, and UP signifies the proportion of rats with ulcers. Conversely, the equation below illustrates the preventive role of treatment.

$$Preventive\;Index(\%)=\frac{\left(UI\;of\;Ug-UI\;of\;Tg\right)}{UI\;of\;Ug}\times 100$$

where UI: ulcer index; Ug: ulcer control; Tg: treated group.

#### 2.6.7. Tissue Collection and Sample Preparation

Tissues of the stomach were dehydrated and each stomach was divided longitudinally and placed on paraffin-coated cardboard for additional investigation, following the macroscopic measurement of the ulcerative index. For immunohistochemical and histopathological studies, one half was preserved in 10% formalin. The other half was standardized in 0.05 M phosphate buffer (pH 7) by a polytron homogenizer at 4 °C for biochemical and immunochemical assessments. The homogenate was applied to centrifugation at 10,000 rpm for a duration of 10 min and the pellet was discarded, while the supernatant representing the cytoplasmic fraction was collected for additional biochemical analyses. An Enzyme-Linked Immunosorbent Assay (ELISA) was utilized to quantify biochemical markers, including GPX, CAT, GSSG, histamine, gastrin, TNF-*α*, and NF-κB. Additionally, a Real-Time Polymerase Chain Reaction was conducted to assess the levels of pERK1/2 and MMP-10. The residual homogenate was stored at −80 °C for subsequent examinations.

#### 2.6.8. Assessment of Oxidative Stress and Inflammatory Biomarkers

Estimation of key biomarkers associated with oxidative stress and inflammation was quantified by using a Sandwich ELISA format. A colorimetric reaction was employed and optical density (OD) was measured in the range 450 to 630 nm.

#### 2.6.9. Determination of Gene Expression by Real-Time Polymerase Chain Reaction (RT-PCR)

The Direct-zol^TM^ RNA Miniprep Plus is an ideal method for purifying high-quality ribonucleic acid (RNA) directly from TRIzol^®^ (tri-reagent isolation solution) samples and is considered an appropriate procedure for various applications like transcription profiling (TP) and a reverse transcription quantitative polymerase chain reaction (RT-qPCR). The SuperScript IV One-Step RT-PCR kit (Cat# 12594100, Thermo Fisher Scientific) was applied for reverse transcription, permitting simultaneous complementary DNA (cDNA) synthesis and polymerase chain reaction (PCR) amplification using gene-specific primers. Relative quantification (RQ) of gene expression was valued for target genes *MMP10* and *pERK1/2*. The RQ was determined using the delta-delta cycle threshold (ΔΔCt) method (Table 1).

**Table 1:** Sequences of primers utilized for the analyzed genes.

	Fo Rward Sequence	Reverse Sequence	Reference
*pERK1/2*	CTCAAGCCTTCCAACCTC	TTCCACGGCACCTTATTT	XM_032899833.1
*MMP10*	GAAGTCCAAGCAGGTTACCC	GTAACACAGCATCAACTTGT	NM_133514.2

#### 2.6.10. Histopathological Evaluation

At the end of the experiment, the glandular stomach was removed and preserved in buffered formalin (10%). The tissues were subjected to dehydration, cleared with xylene, and enclosed in paraffin. For histopathological evaluation, thin sections (3 μm) were prepared and labeled with hematoxylin and eosin (H&E). Additionally, paraffin slices of the glandular stomach were marked with Alcian blue (pH 2.5) to exhibit sulfated mucopolysaccharides [18].

### 2.7. Statistical Analysis

All chemical analyses were performed in triplicate (n = 3) for each experimental group. The results are represented as mean ± standard deviation (SD). Statistical analyses were performed using GraphPad Prism v9.0 (GraphPad Software, Boston, MA, USA). One-way analysis of variance (ANOVA) was used to evaluate statistical differences between groups, following Tukey’s post hoc test, with a significance level at *p* ≤ 0.05.

### 2.8. Molecular Docking Study

The main metabolites detected in the *R. sativus* root extract via UPLC-QTOF-MS/MS were further studied through molecular docking simulations. Molecular Operating Environment (MOE 2015.10) software was used for targeting the active sites of two key proteins: extracellular signal-regulated kinase (ERK; PDB ID: 2H6T) [19] and human matrix metalloproteinase-10 (MMP-10) (PDB ID: 1Q3A) [20]. The Protein Data Bank (PDB) was utilized to obtain the protein structures, which were prepared by eliminating non-essential solvent molecules and cofactors. Additionally, the structural optimization of the remaining residues was performed using standard protocols [13]. To identify the most probable binding pockets, the “Site Finder” tool in the MOE was used. The reliability of these binding sites was verified by re-docking the native co-crystallized ligands. The reference compounds, metabolites MMP-9/10-IN-2 for MMP 10 and MK-8353 for ERK, were protonated, subjected to energy minimization, and formatted into a molecular database (.mdb). Then, docking simulations were performed and the resulting complexes were calculated based on their binding free energy (ΔG a, kcal/mol). The most stable interactions, characterized by the highest ΔG values and root-mean-square deviation (RMSD) values ≤ 2 Å, were visualized in both two-dimensional (2D) and three-dimensional (3D) representations.

## 3. Results

### 3.1. Metabolic Profiling and Molecular Network

**Figure 2:**
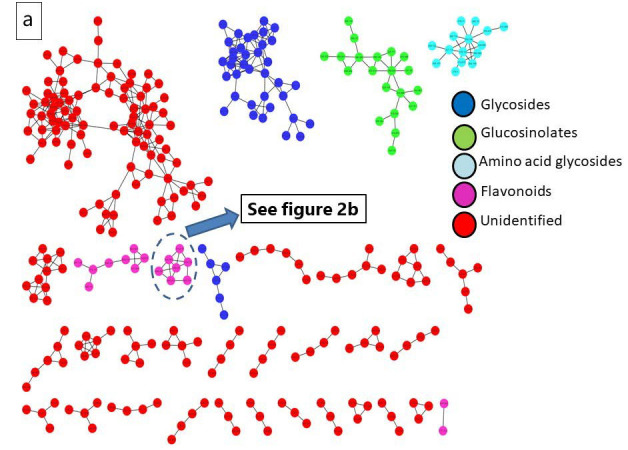

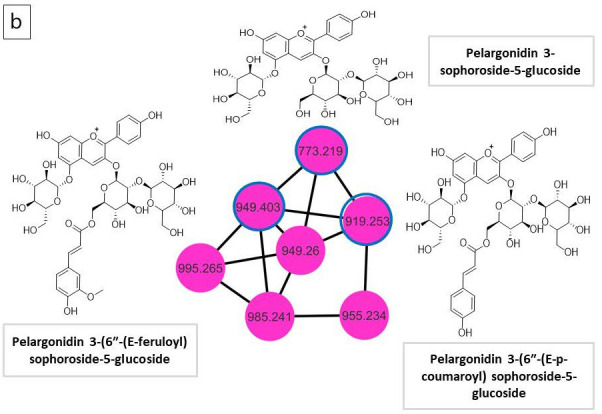
(**a**) Molecular network (MN) of *R. sativus* extract generated from UPLC-ESI–MS in negative ionization mode; (**b**) anthocyanin molecular family network.

A metabolomics analysis of *R. sativus* root extract employing UPLC-QTOF-MS/MS contributed to the recognition of ninety-two bioactive metabolites belonging to multiple phytochemical classes. The major classes include glucosinolates, isothiocyanates, flavonoids, phenolic acids, organic acids, and fatty acids. These compounds were putatively identified by comparing their exact masses with reported values in reputable databases like the Human Metabolome Database (HMDB) and Phytohub, in addition to the relevant scientific literature. The fragmentation patterns of the metabolites were consistent with previously reported studies. The notified information of compounds revealed from *R. sativus* are shown in Table 2, encompassing molecular formula, ontology, name, retention time, mass to charge ratio (*m*/*z*), and mass fragments. The base peak chromatogram of *R. sativus* root ethanolic extract was visualized in (Figure S1). The molecular network (MN) of the plant extract was presented in Cytoscape 3.10.2 for better observation of clusters of compounds including glucosinolates, flavonoids, anthocyanins, and amino acids, as displayed in Figure 2.

#### 3.1.1. Glucosinolates

Amongst the bioactive compounds yielded via *R. sativus*, glucosinolates (GSLs) are the essential phytochemicals in the Brassicaceae family. Their basic structure consists of a *β*-D-thioglucose unit, a sulfonated oxime unit, and a side-chain originating from amino acids [21]. A total of 15 individual GSLs were classified into the three categories, of which ten compounds were assigned to aliphatic GSLs, three were grouped into indolic GSLs, and two were aromatic. Identification of the GSLs relied on the recognition of characteristic product ions, MS/MS fragmentation, retention times, and molecular masses which were used to confirm the identification of the illustrated glucosinolates. GSLs could produce the same and characteristic fragment ions such as *m*/*z* 274.9908, *m*/*z* 259.0139, *m*/*z* 195.0333, *m*/*z* 96.9588, and *m*/*z* 74.9897, which represent (Glu-S-SO_3_^−^), (Glu-SO_4_), (Glu-S^−^), (HSO_4_^−^), and (C2H3SO^−^), respectively. The corresponding selected fragment ion *m*/*z* 96.959 is the most distinct for the identification of GSLs [22]. The mass fragmentation [M−H]^−^ at *m*/*z* 436, fragmented to *m*/*z* 74.9907, 96.9602, 259.0151, and characteristic product ion at *m*/*z* 372.0447 caused by the neutral loss of the methyl sulphoxide moiety, this compound was identified as glucoraphanin [22]. The peak with [M−H]^−^ at *m*/*z* 434.02590 was annotated as glucoraphenin [22]. Sulforaphene, the major isothiocyanate breakdown product from glucoraphanin was detected at *m*/*z* 174.0045 [23]. The [M−H]^−^ at *m*/*z* 372 showed distinctive ion peaks at 96.9605, 195.0321, and 259.0146, and these fragmentations are attributed to gluconapin [24]. Aliphatic glucosinolates at *m*/*z* 406.0297, 386.0572, 420.0446, 434.0603, 388.0333, 416.1046, and 402.0888 with the common significant peaks were identified as Glucoiberverin [22], Glucobrassicanapin [24], Glucoerucin [22], Glucoberteroin [25], Progitrin [25], Heptyl glucosinolate [22], and Hexyl glucosinolate [22], respectively. Three indolic glucosinolate compounds with an even number of nitrogen atoms in their structure were characterized by odd mass fragmentation at *m*/*z* 463.04860, 447.0518, and 477.0638. These compounds were named as 4-hydroxyglucobrassicin [22], glucobrassicin [22], and neoglucobrassicin [22], respectively. The fragmentation of [M−H]^−^ at *m*/*z* 408.0425 showed aromatic glucosinolate identified as glucotropeolin [26]. The definite diagnostic fragment ions were not detected in benzyl glucosinolates such as glucotropeolin, other than the common fragments in the glucosinolates studied [27]. An additional aromatic compound at *m*/*z* 422.0568 with distinct fragment ion 96.9616 was distinguished as gluconasturtiin [28].

#### 3.1.2. Flavonoids

Flavonoids are the main phenolics in the genus *Raphanus*. The different flavonoid subclasses (flavone, flavanone, and flavanols) were also represented by aglycones and glycosides. Four flavanol aglycons were recognized at distinctive peaks at *m*/*z* 317.0308, 315.0505, 301.0349, and 285.0392, which were identified as myricetin [29], isorhamnetin [30], quercetin [31], and kaempferol [30], respectively. Additionally, nine kaempferol glycosides, three quercetin glycosides, three isorhamnetin glycosides, and one methylated flavonol, identified as methylgalangin [30], were detected. The [M−H]^−^ at *m*/*z* 271.0627 was identified by comparison of its exact mass and fragmentation patterns as Naringenin [30]. As well as mass fragmentation, [M−H]^−^ at *m*/*z* 289.0690 was distinguished as Catechin [29].

#### 3.1.3. Anthocyanin

The mass fragmentation at *m*/*z* 287.0551was identified as pelargonidin. The diagnostic peaks at *m*/*z* 449.1079 and 773.2105, corresponding to two non-acylated anthocyanins identified as cyanidin-3-*O*-rhamnoside and pelargonidin 3-sophoroside-5-glucoside, respectively. In addition, two acylated pelargonidin glycosides were recognized at *m*/*z* 919.2529 and 949.2603, which were identified as pelargonidin 3-(6″-(E-p-coumaroyl) sophoroside-5-glucoside and pelargonidin 3-(6″-(E-feruloyl) sophoroside-5-glucoside, respectively. Anthocyanin in the plant can be identified by the presence of the doublet ions of [M–2H]^−^ and [M–2H + H_2_O]^–^, whereas the distinct product ion [M–H]^−^ is predominant for other polyphenols [32].

#### 3.1.4. Phenolic and Organic Acids

Six free Phenolic acids were detected in agreement with the previous literature. The peaks [M-H]^−^ at *m*/*z* 163.0388, 223.0607, and 197.0431 corresponded to *P*-Coumaric acid, Sinapic acid, and Syringic acid, respectively [33]. In comparison with the official database, salicylic acid [33], cinnamic acid [34], and gallic acid [31] were identified with peaks at *m*/*z* 137.0231, 147.0441, and 169.0146, respectively. In addition, sinapoyl malate [35] is a phenolic ester that was recognized at *m*/*z* 339.0728. Numerous organic acids were remarked, including maleic, malic, citric, ascorbic, succinic, malonic, lactic, glucuronic, and pyroglutamic. The flavor profile of different radish varieties is partially influenced by the presence of these acids, which contribute to their unique taste and consumer preference. Lactic acid, malic acid, and succinic acid play significant roles in the unique taste of radishes [36].

#### 3.1.5. Fatty Acids and Their Derivatives

Unsaturated and saturated fatty acids were annotated based on their characteristic mass fragmentation patterns. Unsaturated fatty acids were recognized at *m*/*z* 277.2160, 279.2324, and 281.2474, corresponding to linolenic acid, linoleic acid, and oleic acid, respectively [37]. In contrast, saturated fatty acids were detected at *m*/*z* 227.2004 and 255.2322, which were identified as myristic acid and palmitic acid, respectively [37]. Additionally, fatty acid derivatives were observed, with base peaks at *m*/*z* 295.2272 and 271.2281, corresponding to dimorphecolic acid (9-hydroxy-10, 12-octadecadienoic acid) [38] and Juniperic acid (16-hydroxyhexadecanoic acid) [39], respectively.

#### 3.1.6. Amino Acids and Their Derivatives

They were detected based on the abundant fragment ions of their protonated forms and their respective derivatives. These derivatives resulted either from the loss of H_2_O, yielding their residue mass, or the loss of (H_2_O + CO), forming their corresponding immonium ions. Acidic amino acids included glutamic acid, while basic amino acids comprised histidine, arginine, and lysine. The nonpolar (hydrophobic) amino acids identified were alanine, valine, leucine, phenylalanine, and L-tryptophan. Meanwhile, polar amino acids included threonine, serine, and tyrosine. Additionally, nine acylated amino acids were recognized, as shown in Table 2.

#### 3.1.7. Saccharides

Raffinose, an oligosaccharide, was identified based on its characteristic mass fragmentation, with an assigned peak at *m*/*z* 503.1608. The monosaccharide fructose was annotated based on its fragmentation pattern, showing a peak at *m*/*z* 179.0552. The non-reducing disaccharide sucrose was distinguished by its base peak at *m*/*z* 341.1077. Additionally, the [M−H]^−^ at *m*/*z* 181.0705 was recognized as mannitol, a sugar alcohol.

### 3.2. ZnO-NPs Characterization

#### 3.2.1. UV–Vis Investigation of ZnO-NPs

The maximum spectral peak for synthesized ZnO-NPs was at 363 nm, as displayed in Figure S2. This finding indicates the effective synthesis of ZnO-NPs.

#### 3.2.2. FT-IR Characterization of Synthesized ZnO-NPs and Ethanolic Extract of *R. sativus*

**Figure 3:**
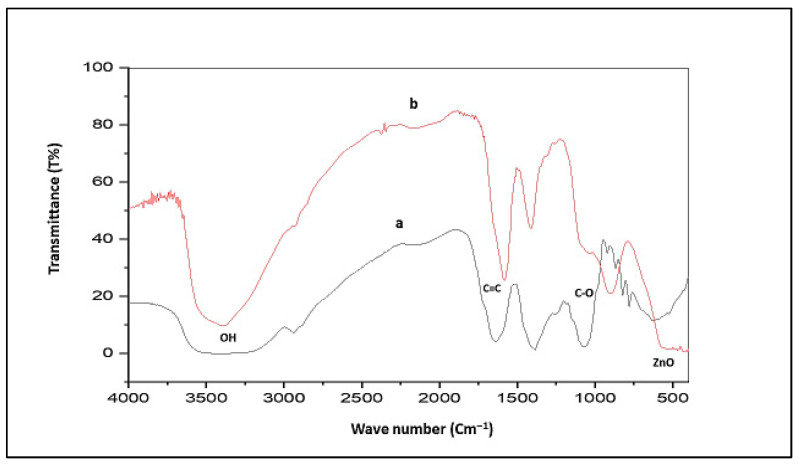
(a) FT-IR spectrum of *R. sativus* extract; (b) FT-IR spectrum of ZnO-NPs.

FT-IR was conducted between 400 and 4000 cm^−1^ to identify the diagnostic functional groups involved in ZnO-NPs biosynthesis mediated by *R. sativus* extract. Concerning *R. sativus*, ethanolic extract (Figure 3a) exhibited a broad absorption at 3356.14 cm^−1^ corresponding to the carboxylic, phenolic, or alcoholic (O-H) groups, whereas the peak at 2939.52 cm^−1^ was associated with the (C-H) stretching vibrations of alkane groups. Additionally, peaks at 2430.31, 2399.45, 2345.44, and 2152.56 cm^−1^ indicated triple-bond stretching. The occurrence of (C=C) stretching of cyclic alkene was identified by the presence of a peak at 1639.49 cm^−1^. A peak at 1384.89 cm^−1^ indicated the incidence of (C-H) stretching of alkanes, where (C-O) stretching of primary alcohols was confirmed by the existence of a peak at 1060.85 cm^−1^. The synthesized ZnO-NPs (Figure 3b) displayed a distinct peak at 3387 cm^−1^ that confirms the incidence of the (O-H) group reliable for H_2_O adsorption on the surface of ZnO-NPs. A peak at 2931.80 cm^−1^ indicated (C-H) stretching of alkanes, as well as a peak at 1581.63 (C-H) cm^−1^ which was representative of the (C=C) stretching of cyclic alkene groups. The prevalence of the alcoholic (O-H) group is ensured by a peak at 1411.89 cm^−1^. The presence of the ZnO stretching band was achieved at 424.34 cm^−1^.

#### 3.2.3. Zeta Potential (ZP) Measurements

**Figure 4:**
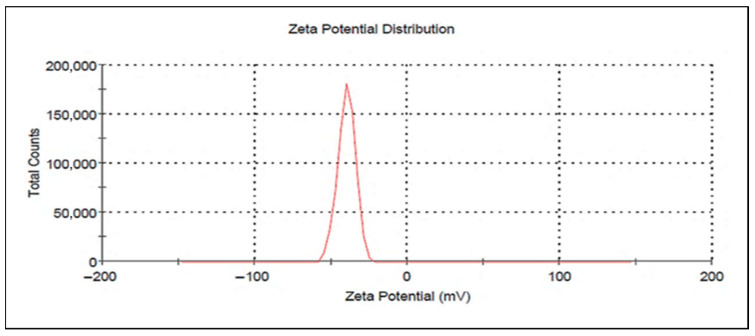
Zeta potential of synthesized ZnO-NPs.

Zeta potential measurements serve as a fundamental indicator for determining ZnO-NPs stability throughout electrophoresis. Figure 4 showed a clear peak at −39.7 mV, with conductivity of 0.310 mS/cm and a zeta deviation of 5.73 mV.

#### 3.2.4. TEM Imaging of ZnO-NPs

**Figure 5:**
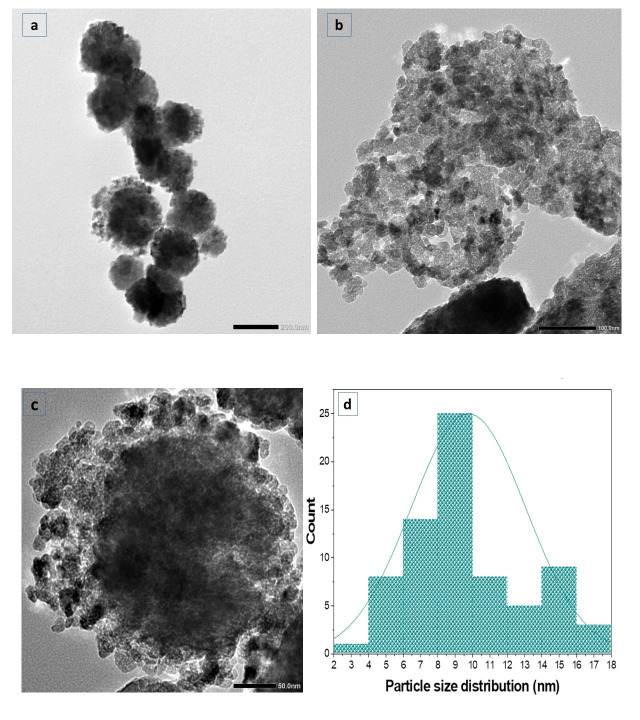
(**a**–**c**) TEM image of ZnO-NPs; (**d**) a histogram of particle size.

TEM was conducted to analyze the particle size and crystalline nature of ZnO-NPs. Figure 5 shows that ZnO-NPs exhibit a hexagonal form, developing cluster aggregates. The size distribution was observed with Image J software (origin 2018), proving a uniform structure, ranging from 17.3 to 4.7 nm, with a mean size of 9.8 nm, and a standard deviation of ±3.3 nm.

#### 3.2.5. X-Ray Diffraction (XRD)

**Figure 6:**
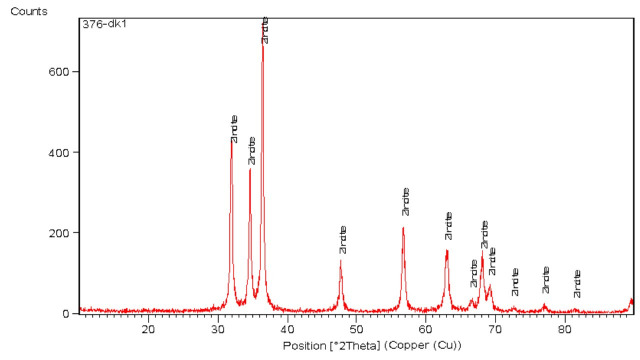
XRD analysis of synthesized ZnO-NPs.

The basic features of ZnO-NPs, including shape, size, and crystal structure, were examined using X-ray diffraction (XRD). The ZnO-NPs synthesized using the ethanolic root extract of *R. sativus* displayed definite peaks, as shown in Figure 6, at 2θ values of 32.0035°, 34.6206°, 36.4363°, 47.7185°, 56.6948°, 62.9485°, 66.5618°, 68.1404°, 69.3007°, 72.6064°, 77.0141°, and 81.6022°. These peaks corresponded with the standard reference pattern from ICDD card 96-900-4179. The findings indicated that the ZnO-NPs exhibited a wurtzite hexagonal crystalline structure.

### 3.3. Biology

#### 3.3.1. LD_50_ Results

Swiss albino mice were orally administrated gradually increasing doses of both the ethanolic extract of *R. sativus* and the synthesized ZnO-NPs, up to 2000 mg/kg body weight. Throughout the 14-day experimental period, all mice were observed closely, particularly during the first 24 hrs following administration. No signs of toxicity or mortality were observed during the initial 24 h or throughout the entire observation period. These findings indicate that the median lethal dose (LD_50_) of both tested samples in mice is estimated to exceed 2000 mg/kg. According to previous reports, substances with an LD_50_ value greater than 50 mg/kg body weight are generally considered safe.

#### 3.3.2. Macroscopic Observations and Evaluation of Ulcer Index, Severity, and Number

**Figure 7:**
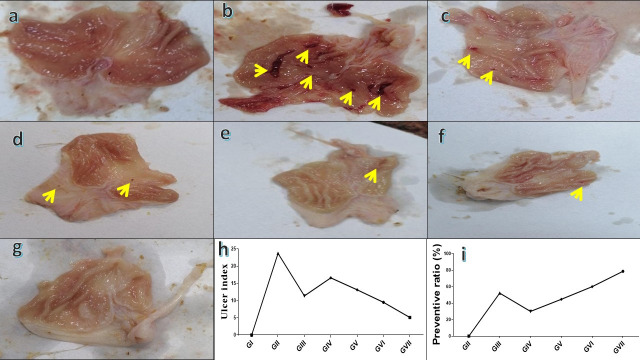
Macroscopic assessment of stomach tissue in rats where (**a**) is a micrograph view of the stomach tissue from group I; (**b**) is a micrograph view of stomach tissue from group II; (**c**) is a micrograph view of the stomach tissue from group III; (**d**) is a micrograph view of the stomach tissue from group IV; (**e**) is a micrograph view of the stomach tissue from group V; (**f**) is a micrograph view of the stomach tissue from group VI; and (**g**) is a micrograph view of the stomach tissue from group VII; (**h**) comparison of ulcer index among experimental groups; (**i**) preventive ratio (%) of the tested treatments. Yellow arrows refer to gastritis/ulcer area.

As shown in Figure 7a, there was no evidence for hemorrhagic lesions or erosions in group I. In contrast, group II (Figure 7b) exhibited a prominent rise in gastric lesions, confirming the successful induction of gastritis. Furthermore, group III, group IV, group V, and group VI (Figure 7c–f) exhibited minimal mucosal injury. Conversely, group VII exhibited normal gastric epithelial lesions, as shown in Figure 7g. The induction of gastritis in group II was further confirmed by a high ulcer index of 23.7 in comparison with the normal control (Figure 7h). Administration of omeprazole (20 mg/kg) significantly reduced the ulcer index to 11.37, corresponding to a protective ratio of 52.04%. Similarly, administration of *R. sativus* extract (100 and 200 mg/kg) lowered the ulcer index to 16.6 and 13.13, achieving preventive ratios of 29.96% and 44.59%, respectively. Remarkably, ZnONP treatment produced a more pronounced gastroprotective effect, reducing the ulcer index to 9.50 and 5.10 at 100 and 200 mg/kg, respectively. At 200 mg/kg, ZnO-NPs showed the highest efficacy, with a preventive ratio of 78.5%, surpassing both the standard drug and the plant extract (Figure 7h,i). Statistical analyses for ulcer severity and ulcer numbers are presented in (Figure S3).

#### 3.3.3. Evaluation of Oxidative Stress Biomarkers (GSSG, GPX, and CAT)

**Figure 8:**
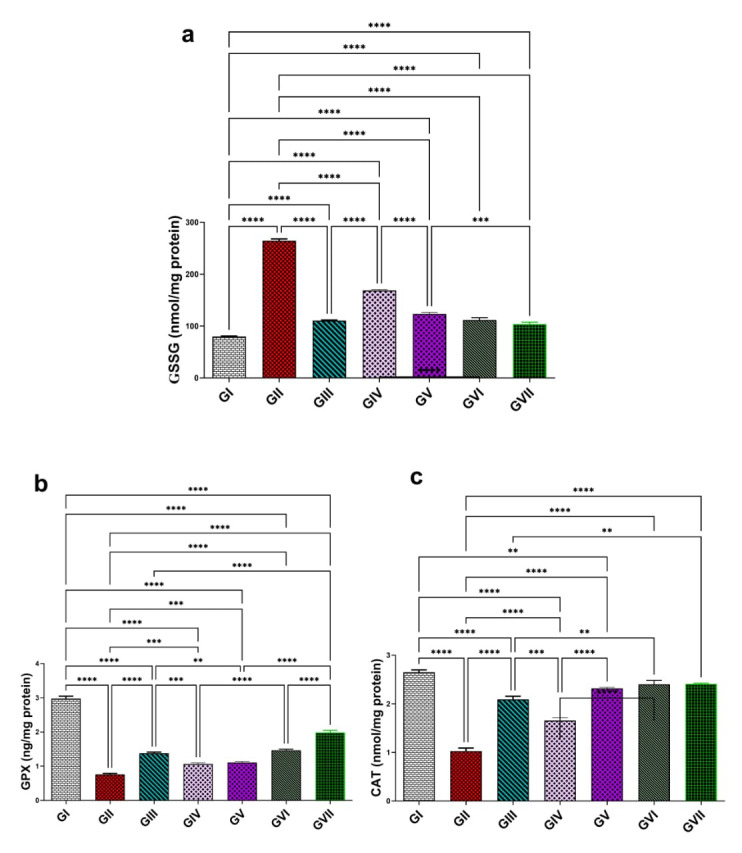
Effect of the root extract of *R. sativus* (100 and 200 mg/kg) and the synthesized ZnO-NPs (100 and 200 mg/kg) on the concentration of GSSG (**a**), GPX (**b**), and CAT (**c**). GI; (control group). GII; (ethanol-induced group). GIII; (omeprazole group 20 mg/kg). GIV; (ethanol–root extract 100 mg/kg). GV; (ethanol–root extract 200 mg/kg). GⅥ; (ethanol–ZnO-NPs 100 mg/kg). GⅦ; (ethanol–ZnO-NPs 200 mg/kg). Results are expressed as mean ± standard error mean (n = 5). Comparisons were made on the basis of the one-way analysis of variance (ANOVA) followed by Tukey’s post hoc test. ** (*p* < 0.01), *** (*p* < 0.001), **** (*p* < 0.0001). GSSG—oxidized glutathione; GPX—glutathione peroxidase; CAT—catalase; ZnO-NPs—zinc oxide nanoparticles.

In comparison to group I, group II had a considerable rise (*p* < 0.0001) in GSSG level (264 ± 7.71), indicating approximately a 230% rise, leading to oxidative damage and impairment of antioxidant scavenging. In contrast, group II showed a significant reduction (*p* < 0.0001) in GPX and CAT levels (0.75 ± 0.068 and 1.00 ± 0.14, respectively), corresponding to decreases of approximately 74.1% and 61.5%, compared with group I (2.9 ± 0.15 and 2.6 ± 0.10, respectively). The oral administration of standard drug, omeprazole, (20 mg/kg) in group III markedly reduced the GSSG concentration (110 ± 2.6, *p*< 0.0001), reflecting a 58.2% reduction relative to group II. On the other hand, group III showed a substantial increase (*p* < 0.0001) in GPX and CAT levels (1.4 ± 0.07 and 2.4 ± 0.18), increases of approximately 96% and 140%, respectively (Figure 8). Rats treated with the extract of *R. sativus* in group IV (100 mg/kg) and group V (200 mg/kg) demonstrated a significant decline (*p* < 0.0001) in GSSG levels (168.70 ± 3.04 and 123.40 ± 6.44) by 36.3% and 53.3%, respectively. Also, ZnO-NPs at 100 and 200 mg/kg in group Ⅵ and group Ⅶ showed a significant decrease (*p* < 0.0001) in GSSG levels (111.60 ± 10.40 and 103.60 ± 8.78), representing 57.8% and 60.8%. Additionally, group IV and group V showed a significant dose-dependent improvement in GPX concentrations by 41.3% (1.06 ± 0.077) and 48% (1.11 ± 0.08), respectively. In addition to a considerable increase in CAT concentration (1.65 ± 0.14 and 2.09 ± 0.14) by 65% and 109%, respectively, relative to group II. ZnO-NPs at levels 100 and 200 mg/kg showed a marked improvement in GPX levels (1.38 ± 0.07 and 1.98 ± 0.14), reflecting advancement of 84% and 164% and in CAT levels (2.3 ± 0.05 and 2.41 ± 0.03) by about 130% and 141%, respectively, compared to group II. The highest ZnONP dose (200 mg/kg) demonstrated the most potent antioxidant potential and superior efficacy among all tested groups. When compared with group III, group Ⅶ exhibited no statically significant difference (a modest 6.2% reduction), but exhibited a significant increase in CAT levels (0.42%, *p* < 0.01) as well as a marked increase (34.7%, *p* < 0.0001) in GPX concentration. All results are represented in Figure 8.

#### 3.3.4. Assessment of Gastric Secretions and Inflammatory Biomarkers (Gastrin, Histamine, TNF-α, and NF-κB)

**Figure 9:**
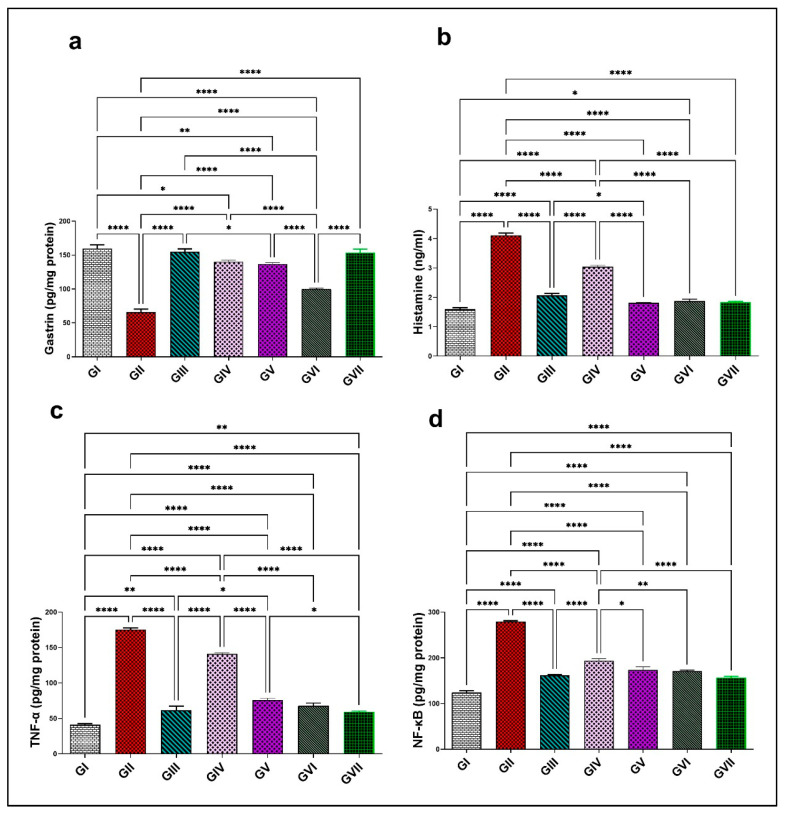
Effect of the root extract of *R. sativus* (100 and 200 mg/kg) and the synthesized ZnO-NPs (100 and 200 mg/kg) on the concentration of gastrin (**a**), histamine (**b**), TNF-*α* (**c**), and NF-κB (**d**). Group I; (negative control group). Group II; (ethanol-induced group). Group III; (ethanol–omeprazole 20 mg/kg). Group IV; (ethanol–root extract 100 mg/kg). Group V; (ethanol–root extract 200 mg/kg). Group Ⅵ; (ethanol–ZnO-NPs 100 mg/kg). Group Ⅶ; (ethanol–ZnO-NPs 200 mg/kg). Results are expressed as mean ± standard error mean (n = 5). Comparisons were made on the basis of the one-way analysis of variance (ANOVA) followed by Tukey’s post hoc test. * (*p* < 0.05), ** (*p* < 0.01), **** (*p* < 0.0001). ZnO-NPs—zinc oxide nanoparticles; TNF-α—tumor necrosis factor alpha; NF-κB—nuclear factor kappa B.

A notable decline in gastrin hormone levels was noted in group II (65.7 ± 10.2) relative to group I (159 ± 12.8), with a decrease of 58.7%. Conversely, histamine, TNF-*α*, and NF-κB exhibited a significant elevation (4.1 ± 0.17, 175.1 ± 5.97, and 279.3 ± 4.8) with a ratio of 156.3% for histamine, 324.5% for TNF-*α*, and 124.6% for NF-κB, compared with group I (1.6 ± 0.13, 41.25 ± 3.2, and 124.3 ± 9.4), respectively. Treatment with omeprazole 20mg/kg in group III demonstrated a substantial elevation (*p* < 0.0001) in gastrin concentration (153.45 ± 11.97, *p* < 0.0001) by a 134% rise, as shown in Figure 9a, while it effectively decreases (*p* < 0.0001) histamine (1.8 ± 0.075), TNF-*α* (61.35 ± 13.6), and NF-κB levels (161.95 ± 4.31), by 56.1%, 65%, and 42%, respectively, in comparison to group II, as displayed in Figure 9. Groups from IV to group Ⅶ showed a significant elevation in gastrin levels with an increase in efficacy at higher doses (*p* < 0.0001), with values of 100 ± 2.9, 136.9 ± 4.3, 140 ± 5.7, and 154.95 ± 9.3. These represent increasing ratios of 52.3%, 108%, 113.2%, and 136%, respectively, in comparison to group II. In contrast, these groups displayed a marked inhibition (*p* < 0.0001) in histamine concentration (3 ± 0.1, 2.1 ± 0.15, 1.87 ± 0.15, and 1.8 ± 0.03) by percentages of 26.8%, 48.8%, 54.4%, and 56.1%, respectively. Likewise, TNF-α levels decreased (*p* < 0.0001) to 141 ± 3.64, 75.9 ± 5.85, 67.8 ± 8.38, and 59.45 ± 2.33 by approximately 19.4%, 56.6%, 61.3%, and 66.1%, respectively. NF-κB levels were significantly suppressed (*p* < 0.0001) in the similar groups, measuring 194.1 ± 9.24, 173.9 ± 15.29, 170.9 ± 5.21, and 156 ± 7.9 and approaching 30.5%, 37.7%, 38.8%, and 44.15%, respectively, in comparison to group II. ZnO-NPs at 200 mg/kg and omeprazole both significantly improved the level of gastrin and successfully restored the levels of histamine, TNF-*α*, and NF-κB proximate to the control levels. Both groups did not differ significantly, even though ZnO-NPs at 200 mg/kg showed a non-significant rise in efficacy, further reinforcing its potential as an alternative to the reference drug. All findings are demonstrated in Figure 9.

#### 3.3.5. Assessment of MMP-10 and pERK1/2 by RT-PCR

**Figure 10:**
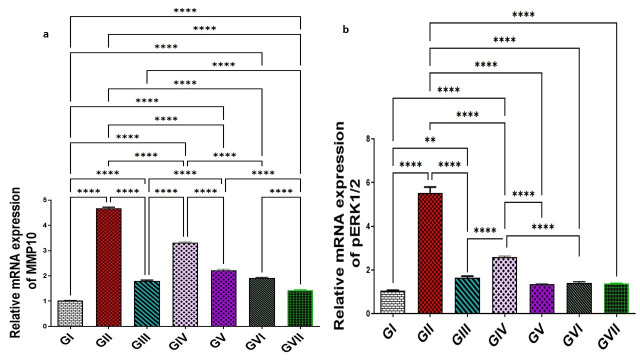
Effect of the root extract of *R. sativus* (100 and 200 mg/kg) and the synthesized ZnO-NPs (100 and 200 mg/kg) on the concentration of MMP-10 (**a**) and pERK1/2 (**b**). Group I; (negative control group). Group II; (ethanol-induced group). Group III; (ethanol–omeprazole 20 mg/kg). Group IV; (ethanol–root extract 100 mg/kg). Group V; (ethanol–root extract 200 mg/kg). Group Ⅵ; (ethanol–ZnO-NPs 100 mg/kg). Group Ⅶ; (ethanol–ZnO-NPs 200 mg/kg). Results are expressed as mean ± standard error mean (n = 5). Comparisons were made on the basis of the one-way analysis of variance (ANOVA) followed by Tukey’s post hoc test. ** (*p* < 0.01), **** (*p* < 0.0001). ZnO-NPs—zinc oxide nanoparticles; MMP-10—matrix metalloproteinase-10; pERK1/2—phosphorylated extracellular signal-regulated kinase 1/2.

In group II, the expression levels of MMP-10 and pERK1/2 were significantly increased (*p* < 0.0001), showing elevations (4.68 ± 0.089 and 5.54 ± 0.58) representing 360.6% and 423.6%, respectively, compared with group I (1.016 ± 0.01 for MMP-10 and 1.06 ± 0.05 pERK1/2). All treatment groups, including the standard drug (omeprazole), the extract (100 and 200 mg/kg), and ZnO-NPs at both dose levels (100 and 200 mg/kg), could significantly downregulate (*p* < 0.0001) both MMP-10 and pERK1/2 compared to group II. MMP-10 declined to 1.79 ± 0.09, 3.32 ± 0.057, 2.22 ± 0.09, 1.91 ± 0.08, and 1.43 ± 0.05, corresponding to a reduction of 61.8%, 29.1%, 52.3%, 59.2% and 69.4%, respectively. Moreover, pERK1/2 levels were reduced to 1.38 ± 0.034, 2.61 ± 0.09, 1.66 ± 0.14, 1.41 ± 0.12, and 1.37 ± 0.04 by percentages of 75.1%, 52.9%, 70%, 74.5%, and 75.3%, respectively. Notably, ZnO-NPs (200 mg/kg) demonstrated significantly superior efficacy (*p* < 0.0001) over omeprazole in downregulating MMP-10 expression by an additional 7.6%. However, no significant differences were observed between ZnO-NPs (200 mg/kg) and omeprazole in reducing pERK1/2 expression levels. All data are shown in (Figure 10).

#### 3.3.6. Microscopic Examination

**Figure 11:**
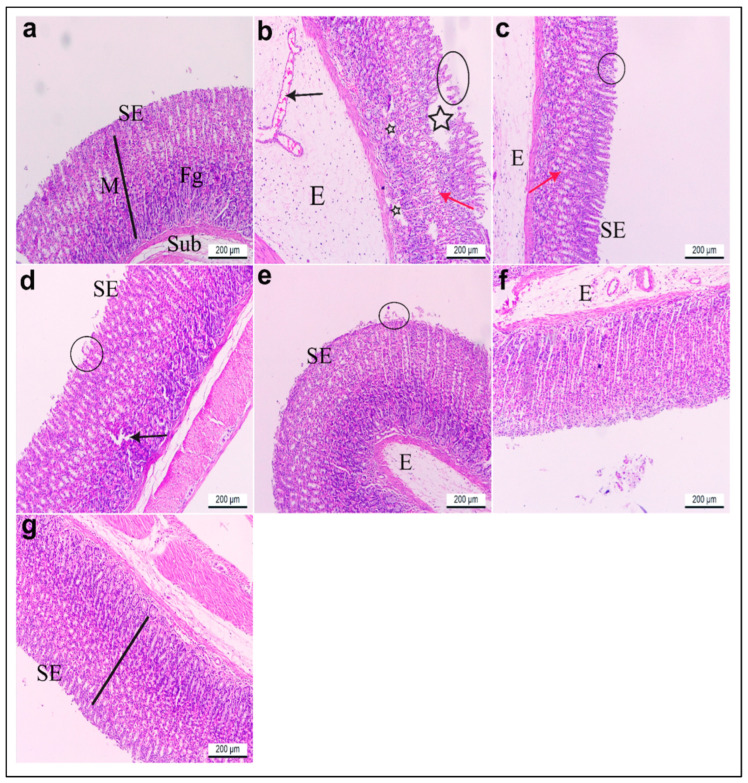
(**a**–**g**) Glandular stomach sections of adult rats. H&E stain. ×100. (**a**) Group I had intact gastric mucosa (M) (black line) with normal lining surface epithelium (SE), normal fundic glands (Fg), and normal tunica submucosa (Sub). (**b**) Group II showed severe edema (E), congested and dilated blood capillaries (black arrow) in the submucosal layer, loss of surface epithelium (black circle), deep ulcerative patches (black stars) reaching the lamina propria, and degenerated fundic glands (red arrow). (**c**) Group III revealed a nearly normal lining epithelium (SE), less edema (E) in the submucosa, and nearly normal-shaped fundic glands (red arrow) except for a few parts of the mucosa with detached surface epithelia (black circle). Gastritis rats pretreated with (**d**) low (group IV) and (**e**) high (group V) doses of *R. sativus* extracts showed preservation of the gastric mucosa lining epithelium (SE), except for a few detached surface epithelia (black circle), some degenerated fundic glands (black arrow), and less edema (E). (**f**) Gastritis rats pretreated with a low (group VI) dose of synthesized ZnO-NPs showed a preserved gastric mucosa histoarchitecture and lining epithelium (SE) except for a few edemas (E). (**g**) Gastritis rats pretreated with a high (group VII) dose of synthesized ZnO-NPs displayed nearly gastric mucosa with intact surface epithelium (SE).

Sections of the glandular stomach from the rats in group I stained with hematoxylin and eosin (H&E) exhibited an intact gastric mucosa with a well-preserved surface columnar epithelium. The lamina propria contained fibroelastic connective tissue and straight fundic glands, while the tunica submucosa consisted of loose connective tissue (Figure 11a). Gastric tissue from group II showed significant histopathological damage compared to group I. Observed alterations included severe edema, congestion, and dilation of blood capillaries within the submucosal layer. Additionally, there was a notable loss of surface epithelium, deep ulcerative lesions extending to the lamina propria, and degeneration of the fundic glands (Figure 11b). Rats in group III exhibited considerable restoration of gastric mucosal structure. The surface epithelium appeared mostly intact with reduced edema in the submucosa and nearly normal-shaped fundic glands. However, some areas still displayed minor detachment of surface epithelium (Figure 11c). Likewise, rats pretreated with low and high doses of *R. sativus* extract in groups IV and V demonstrated notable preservation of the gastric mucosal lining. While some areas showed mild epithelial detachment and partially degenerated fundic glands, edema was significantly reduced compared to the ulcerative group (Figure 11d,e). Furthermore, treatment with low and high doses of synthesized ZnO-NPs in groups VI and VII demonstrated a marked preservation of the gastric mucosa histoarchitecture, which appeared nearly normal upon increasing the dose compared to the ulcerative group, except for the presence of a few edema in the submucosa in group VI that disappeared by pretreatment with a high dose of synthesized ZnO-NPs (Figure 11f,g).

#### 3.3.7. Histochemical Investigations of Alcian Blue pH (2.5)

**Figure 12:**
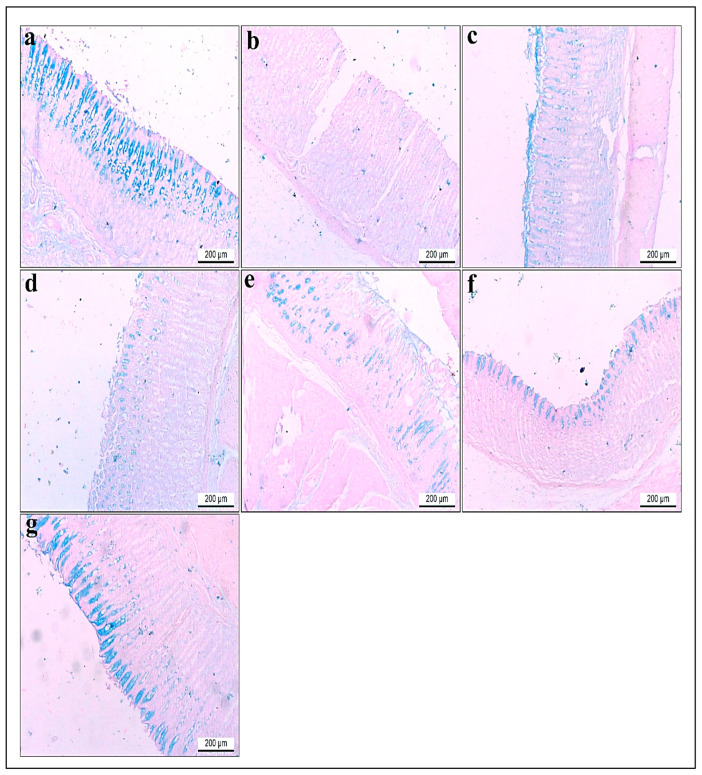
(**a**–**g**) Glandular stomach sections of adult rats. Alcian blue stain pH (2.5). ×100. (**a**) Group I had intense acidophilia in the lining of the mucosa. (**b**) Group II showed faint blue staining to Alcian blue compared to control rats. (**c**) Group III revealed moderate staining to Alcian blue compared to group II. (**d**) Gastritis rats pretreated with a low dose of *R. sativus* extract in group IV showed mild reactions to Alcian blue. (**e**) Gastritis rats pretreated with a high dose of *R. sativus* extract in group V revealed a moderate reaction to Alcian blue. (**f**) Gastritis rats pretreated with a low dose of synthesized ZnO-NPs in group VI demonstrated moderate staining to Alcian blue compared to group II. (**g**) Gastritis rats pretreated with a high dose of synthesized ZnO-NPs in group VII revealed intense acidophilia compared to group II.

Histological analysis of gastric tissue using Alcian blue staining revealed distinct differences among the experimental groups. The gastric tissue in group I stained with Alcian blue revealed intense acidophilia in the lining of the mucosa (Figure 12a). Contrariwise, the gastric tissue of rats in group II showed faint blue staining to Alcian blue that significantly decreased by 0.2 compared to control rats in group I (Figure 12b). On the other hand, gastric sections in group III displayed moderate staining with Alcian blue, which increased significantly by 8.5 compared to group II (Figure 12c). Meanwhile, the gastric tissue of gastritis rats pretreated with a low dose of root extract in group IV revealed a mild reaction to Alcian blue with a significant increase in acidophilia by 3.9 compared to group II (Figure 12d). However, the gastric tissue of rats with a higher dose of root extract in group V and low dose of synthesized ZnO-NPs in group VI showed moderate reactivity with significantly elevated acidophilia by 5.8 and 6.5, respectively, compared to group II (Figure 12e,f). Furthermore, the gastric tissue of rats with a higher dose of synthesized ZnO-NPs in group VII demonstrated significant restoration of the lining mucosa and increased acidophilia by 9.4, compared to group II (Figure 12g). All findings are displayed in (Table 3 and Figure 12).

#### 3.3.8. Correlation Analysis of the Key Parameters

**Figure 13:**
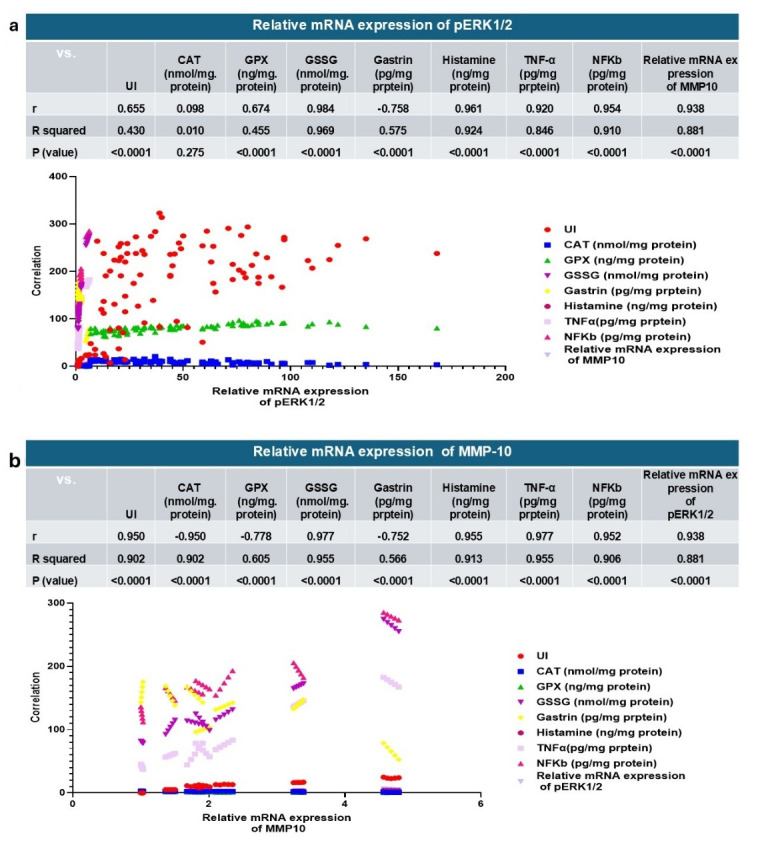
(**a**): relative mRNA expression of pERK1/2, (**b**): and relative mRNA expression of MMP-10 correlated to the other gastroprotective effect and the key parameters. CAT—catalase; GPX—glutathione peroxidase; GSSG—oxidized glutathione; TNF-α—tumor necrosis factor alpha; NF-κB—nuclear factor kappa B; MMP-10—matrix metalloproteinase-10; pERK1/2—phosphorylated extracellular signal-regulated kinase 1/2.

Correlation studies provide additional insights into how the main parameters relate to each other and strengthen the interpretation of the treatment’s efficacy. Strong positive correlation was observed between pERK1/2 and several parameters, including (UI, CAT, GPX, GSSG, histamine, TNF-α, NF-κB, and MMP-10) with correlation coefficients (r) (0.655, 0.098, 0.674, 0.984, 0.961, 0.920, 0.954, and 0.938, respectively), while gastrin had a negative correlation with pERK1/2 with an r value of −0.758, as shown in Figure 13a. Otherwise, there were negative and positive correlations between the main parameters and MMP-10. The negative correlations were recorded for CAT, GPX, and gastrin with r values of −0.950, −0.778, and −0.752, respectively. Positive correlations were recorded for UI, GSSG, histamine, TNF-*α*, NF-κB, and pERK1/2 with r values equal to 0.950, 0.977, 0.955, 0.977, 0.952, and 0.938, respectively, as presented in Figure 13b. These findings suggest that the activation of pERK1/2 and MMP-10 is closely linked to gastric mucosal injury, as exhibited by their strong positive association with ulcer index, pro-inflammatory mediators (TNF-α, NF-κB, and histamine), and oxidative stress markers (GSSG). The negative correlations with antioxidant enzymes (CAT and GPX) indicate that the depletion of the antioxidant defense system contributes to ERK/MMP-10, signaling activation during gastritis. The inverse relationship between gastrin and both pERK1/2 and MMP-10 implies a protective role of gastrin in maintaining gastric mucosal integrity, possibly through modulation of acid secretion and mucosal repair mechanisms. These correlations provide further support for the therapeutic potential of targeting the ERK1/2–MMP-10 axis in disease attenuation.

### 3.4. Docking Simulations

**Figure 14:**
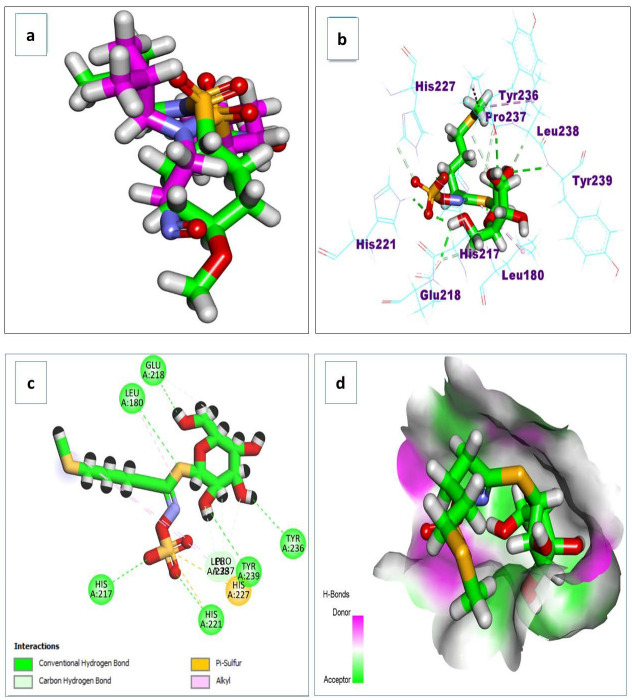
(**a**) The redocking of the co-crystallized ligand; (**b**) 3D binding mode; (**c**) 2D binding mode; and (**d**) surface map of the glucoerucin on the active site of the matrix metalloproteinase 10 (PDB ID: 1Q3A).

**Figure 15:**
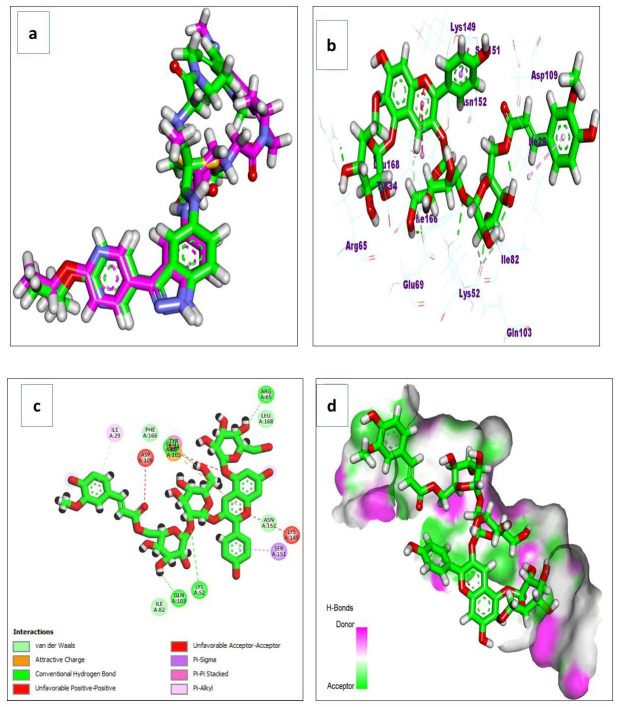
(**a**) The redocking of the co-crystallized ligand; (**b**) 3D binding mode; (**c**) 2D binding mode; and (**d**) surface map of the pelargonidin 3-6″-(E-feruloyl) sophoroside-5-glucoside on the active site of the ERK (PDB ID: 6DCG).

Docking simulations on the major metabolites identified in *R. sativus* root extract suggest their potential as natural gastroprotective agents, primarily through the inhibition of matrix metalloproteinase-10 (MMP-10) and extracellular signal-regulated kinase (ERK), key enzymes implicated in gastritis-associated inflammation, tissue remodeling, and mucosal injury. After validating the docking protocol (Figure 14a), the screened compounds demonstrated strong binding affinities (ΔG a) to MMP-10, ranging from −4.13 to −11.27 kcal/mol (Table 4). Notably, glucosinolates exhibited superior binding, with glucoerucin (ΔG a = −11.27 kcal/mol) outperforming the reference inhibitor (ΔG a = −6.04 kcal/mol). Interaction analysis revealed that glucoerucin stabilizes within MMP-10′s collagenase active site via multiple hydrogen bonds: three between its sugar moiety hydroxyls (Glu218, Tyr236, and Tyr239) and two between its sulfate group and the catalytic residues His217 and His221. Additionally, the isothiocyanate sulfur formed a hydrogen bond with Leu180. For ERK, the compounds showed binding affinities between −5.33 and −10.90 kcal/mol (Table 4), with glycosylated/acylated anthocyanins and flavonoids displaying notable activity. Pelargonidin 3-(6″-(E-feruloyl) sophoroside-5-glucoside exhibited the highest affinity (ΔG a = −10.90 kcal/mol), approaching that of the reference compound (ΔG = −12.00 kcal/mol). Binding mode analysis in Figure 15 highlighted hydrogen bonds between the sugar hydroxyls and key ERK residues (Lys52, Arg65, Glu69, and Gln103), further supporting their inhibitory potential.

## 4. Discussion

*R. sativus* has a potential therapeutic effect and represents a promising natural cure for gastritis and peptic ulcers. Purple radish (*R. sativus* L.) extract showed gastroprotection in ethanol-injury models in rats [40]. In ulcerative colitis models, *R. sativus* seed extract ameliorates intestinal oxidative and inflammatory damage, supporting its utility for the management of intestinal disorders [41]. Similarly, fresh juice from the plant has anti-gastric ulcer potential, attributed to its phenolics and sulfur-containing compounds, which act by eliminating free radicals and preserving the stomach tissue [6]. These findings support the folkloric use of the plant in gastropathy [6].

Our metabolomic study of the plant extract revealed the existence of potential bioactive metabolites, such as glucosinolates, isothiocyanates, flavonoids, fatty acids, amino acids, phenolic acids, organic acids, and saccharides. Sulforaphane, a well-known isothiocyanate originating from its precursor glucoraphanin, has bactericidal abilities against *H. pylori* [42]. These bacteria are broadly associated with chronic gastritis, ulcers, and gastric cancer. The potential use of Brassicaceae-rich food is as adjunctive therapy with antibiotics for *H. pylori* eradication, offering synergistic activity and possibly reducing antibiotic side effects [43]. Also, flavonoids have therapeutic potential for the management of gastrointestinal disorders. They inhibited acid production in isolated parietal cells in response to various inflammatory biomarkers and stimulated prostaglandin E2 production in isolated gastric mucosal cells associated with *H. pylori* infection [44]. Flavonoids exhibit an important defense role against oxidative damage via preventing the generation of reactive oxygen species and scavenging free radicals [45].

The successful synthesis of ZnO-NPs was verified using several characterization methods. UV–Vis showed a maximum absorption peak at 363 nm, indicating the formation. TEM analysis exposed small aggregate particles with hexagonal crystal features, and XRD patterns confirmed a hexagonal crystalline arrangement of the wurtzite type [46,47]. Moreover, FT-IR further validated the formation of ZnO-NPs and zeta potential established their stability [48]. These findings demonstrate the formation, stability, and integrity of ZnO-NPs, consistent with previous studies [49].

Ethanol-induced gastric ulcer is closely related to oxidative stress, which is mediated by free oxygen radicals. One of our targets was to determine the antioxidant potential of the *R. sativus* plant. The elevation of glutathione disulfide (GSSG) levels in gastric tissue, the oxidized form of glutathione, is linked to mucosal inflammation in animal models [50]. Also, the inhibition in glutathione peroxidase (GPX) activity is noticed in gastritis, revealing impairment in the cellular defense mechanism and an increase in gastric injury. Likewise, catalase (CAT) concentrations decreased in gastritis, causing accumulation of toxic hydrogen peroxide and worsening inflammation. In consequence, the disruption in the balance of these antioxidant biomarkers is accompanied by oxidative stress, which contributes to gastric mucosal injury. Histamine, along with gastrin and acetylcholine, impacts the process of gastric secretions [51]. Tumor Necrosis Factor-Alpha (TNF-*α*) is the key pro-inflammatory cytokine induced in apoptosis, oxidative stress, and involved in disease progression [52]. Furthermore, Nuclear Factor Kappa B (NF-κB) is frequently triggered in gastritis under the conditions of *Helicobacter pylori* infection and oxidative stress [53]. Matrix metalloproteinase-10 (MMP-10) plays a critical role in inflammation and tissue damage and is closely linked with the activation of NF-κB. Phosphorylated extracellular signal-regulated kinases 1 and 2 (pERK1/2), important key factors of the mitogen-activated protein kinase (MAPK) pathway, amplify inflammation and trigger epithelial cell damage.

In the toxicity assessment, both the *R. sativus* root extract and ZnO-NPs were safe up to 2000 mg/kg in mice. In our study, ethanol induced significant mucosal damage in rats. Conversely, the treatment with the standard drug (omeprazole), *R. sativus* root extract, and ZnO-NPs reduced both the number and severity of ulcers. ZnO-NPs, at a dose of 200 mg/kg, showed the most potent gastroprotective effect among the tested groups. Our paper exhibits that ethanol administration-induced gastric ulcers in rats are strongly related to oxidative stress and inflammatory responses; the elevation in the level of oxidized glutathione (GSSG) and the decrease in the activities of key enzymes, involving glutathione peroxidase (GPX) and catalase (CAT); and also, the disruption in gastric secretory functions including suppressed gastrin levels and elevated inflammatory mediators like histamine, TNF-α, and NF-κB. Additionally, ethanol exposure causes upregulated expression of MMP-10 and pERK1/2, which are associated with tissue inflammation and damage. Collectively, these findings are indicative of compromised cellular antioxidant defenses and increased oxidative damage within gastric tissue.

Treatment with both *R. sativus* extract and ZnO-NPs effectively alleviated ethanol-induced gastric injury, showing a significant dose-dependent restoration of antioxidant balance, improvement in gastrin secretion, and reduction in pro-inflammatory markers. ZnO-NPs at a dose of 200 mg/kg displayed the most potent antioxidant effect and exhibited strong anti-inflammatory activity exceeding the effect of the reference drug (omeprazole). This highlights their powerful role in enhancing cellular defense mechanisms, promoting mucosal healing, modulating inflammatory signaling pathways, and contributing to tissue protection and repair. Histopathology examination supported these findings, revealing extensive gastric tissue damage in the ethanol-induced group, including epithelial injury, submucosal edema, and glandular degeneration. The treatment groups, specifically those receiving ZnO-NPs (200 mg/kg), exhibited a preserved mucosal structure and reduced pathological changes. Furthermore, histochemical analysis approved the protective effect, with all treated groups showing repair of acidic mucins essential for mucosal integrity.

A molecular docking study of the major metabolites from plant extract revealed strong inhibitory potential to gastritis and mucosal damage, particularly glucosinolates, flavonoids, and anthocyanins. Generally, these outcomes highlight the potent therapeutic potential of the green-synthesized ZnO-NPs derived from *R. sativus* in protecting against ethanol-induced gastric injury. In recent studies, the anti-inflammatory activity and biomedical effect of green-synthesized ZnO-NPs using plant extract further support their antioxidant potential observed in the current work [54,55]. ZnO-NPs represent an effective, safe, and natural promising therapeutic strategy for the management of gastritis due to their potent anti-inflammatory, antioxidant, and gastro-protective influences. The sustainable approach improved the biological compatibility and safety of ZnO-NPs and enriched them with natural phytochemical compounds present in *R. sativus* that synergistically contribute to mucosal healing. The greater therapeutic influence of these nanoparticles may be attributed to the presence of phytochemicals in *R. sativus*, such as phenolics and sulfur-containing compounds, which act as reducing and stabilizing agents during nanoparticle synthesis and are known for their strong antioxidant and anti-inflammatory activities [15]. Future studies are needed to elucidate the mechanisms underlying their gastroprotective properties and to validate their therapeutic potential through clinical trials.

## 5. Conclusions

The present study provides a first report on the ecofriendly formation of ZnO-NPs via *R. sativus* and their promise for managing gastritis and protecting against gastric mucosal damage. The metabolic analysis identifies numerous bioactive compounds, including glucosinolates, isothiocyanates, and flavonoids, which have antioxidant and anti-inflammatory efficacy. Treatment with *R. sativus* root extract and ZnO-NPs significantly restored antioxidant enzyme activity (GPX and CAT), diminished oxidative damage (GSSG), and attenuated the expression of biomarkers (TNF-*α*, NF-κB, MMP-10, and pERK1/2). These alterations were associated with an improvement in gastrin secretion, decreased histamine concentrations, and noticeable protection of gastric mucosal integrity, which was confirmed by histochemical and histological studies. Notably, ZnO-NPs at a higher dose (200 mg/kg) demonstrated superior efficacy to the crude extract and sometimes superior efficacy to the standard drug, omeprazole. These outcomes highlight the promising use of the green-synthesized ZnO-NPs as an eco-friendly, effective, and natural therapeutic candidate for the prevention and management of gastritis and related gastric mucosal injuries. Future research should focus on clinical investigation, dosage optimization, and long-term safety to completely establish their biomedical potential.

## Figures and Tables

**Table 2:** Characterization of secondary metabolites in the *R. sativus* via LC–ESI-TOF–MS:

No.	RT (min)	Molecular ion (*m*/*z*)^−^	Metabolite Name	Formula	Fragmentation	Error PPM
Glu cosinolates						
1.	1.13	434.0259	Glucoraphenin	C_12_H_21_NO_10_S_3_	96.9598	3.5
2.	1.15	434.0623	Glucoberteroin	C_13_H_25_NO_9_S_3_	96.9598	3.5
3.	1.16	436.0399	Glucoraphanin	C_12_H_23_NO_10_S_3_	372.0447, 259.0151, 96.9602, 74.9907	0.3
4.	1.45	463.0486	4-Hydroxyglucobrassicin	C_16_H_20_N_2_O_10_S_2_	96.9598	2.2
5.	1.8	372.0443	Gluconapin	C_11_H_19_NO_9_S_2_	259.0146, 195.0321, 96.960	6.9
6.	1.85	406.0297	Glucoiberverin	C_11_H_21_NO_9_S_3_	258.9979, 96.9612, 74.9916	0.6
7.	2.11	386.0572	Glucobrassicanapin	C_12_H_21_NO_9_S_2_	258.9398, 112.9844, 96.9607	0.5
8.	2.00	408.0425	Glucotropeolin	C_14_H_19_NO_9_S_2_	232.0593, 164.0716	1.8
9.	2.23	420.0446	Glucoerucin	C_12_H_23_NO_9_S_3_	259.0095, 96.9607, 74.9907	1.2
10.	2.80	447.0518	Glucobrassicin	C_16_H_20_N_2_O_9_S_2_	294.8948, 96.9575	1.7
11.	3.78	422.0568	Gluconasturtiin	C_15_H_21_NO_9_S_2_	96.9616	1.4
12.	4.16	477.0638	Neoglucobrassicin	C_17_H_22_N_2_O_10_S_2_	96.9607, 74.9913	1.2
13.	5.66	174.0045	Sulforaphene	C_6_H_9_NOS_2_	158.9815, 130.9672, 57.9743	1.8
14.	6.18	388.0333	Progitrin	C_11_H_19_NO_10_S_2_	258.9395, 195.0306, 96.9596	8.7
15.	6.21	416.1046	Heptyl glucosinolate	C_14_H_27_NO_9_S_2_	96.9601	0.6
16.	8.66	402.0888	Hexyl glucosinolate	C_13_H_25_NO_9_S_2_	96.9693, 274.8984	0.2
Flavonoids						
17.	1.70	289.0690	Catechin	C_15_H_14_O_6_	243.0623	5.8
18.	1.78	283.0624	Methylgalangin	C_16_H_12_O_5_	240.0451, 146.9627	8.1
19.	2.54	317.0308	Myricetin	C_15_H_10_O_8_	248.9649, 180.9708, 112.9858	5.1
20.	2.89	271.0627	Naringenin	C_15_H_12_O_5_	203.0830, 116.0524	9.6
21.	3.73	771.1962	Kaempferol 3-*O*-sophoroside-7-glucoside	C_33_H_40_O_21_	609.1463, 285.0451	2.1
22.	4.15	933.2294	Quercetin 3-*p*-coumarylsophoroside-7-glucoside	C_42_H_46_O_24_	771.1764	0.1
23.	4.63	977.2547	Kaempferol 3-(2″′-sinapoylsophoroside) 7-glucoside	C_44_H_50_O_25_	815.2082	1.1
24.	4.79	947.2460	Kaempferol 3-*O*-feruloyl-sophoroside 7-*O*-glucoside	C_43_H_48_O_24_	785.1880	0.9
25.	4.84	639.1525	Dactylin	C_28_H_32_O_17_	477.1005, 315.0506	4.8
26.	5.40	609.1432	Rutin	C_27_H_30_O_16_	404.8315, 301.0359	2.8
27.	5.55	431.0984	Kaempferol-3-*O*-rhamnoside	C_21_H_20_O_10_	385.1902, 285.0259, 278.9101	2.6
28.	5.65	755.2040	Kaempferol 3-*O*-glucosyl-rhamnosyl-glucoside	C_33_H_40_O_20_	593.1487, 447.0902, 285.0391	1.4
29.	6.07	593.1505	Nicotiflorin	C_27_H_30_O_15_	447.0943, 431.0976, 285.0392	0.7
30.	6.13	623.1607	Narcissin	C_28_H_32_O_16_	315.0504, 299.0191	0.1
31.	6.25	739.2100	Kaempferol 3-*O*-xylosyl-rutinoside	C_33_H_40_O_19_	593.1396, 431.0936, 285.0400	2.7
32.	6.28	463.0867	Isoquercitrin	C_21_H_20_O_12_	218.9489, 286.9362, 300.0263	0.9
33.	6.64	577.1540	Kaempferitrin	C_27_H_30_O_14_	285.0409, 431.0968	2
34.	6.73	315.0505	Isorhamnetin	C_16_H_12_O_7_	152.0071, 188.9387, 246.8939	1.7
35.	6.83	447.0954	Astragalin	C_21_H_20_O_11_	112.9871, 284.0353, 285.0452	7.2
36.	7.04	477.1031	Isorhamnetin-3-*O*-glucoside	C_22_H_22_O_12_	314.0437, 243.0307	0.7
37.	9.23	301.0349	Quercetin	C_15_H_10_O_7_	121.0315, 151.0050, 255.2376	2.1
38.	10.48	285.0392	Kaempferol	C_15_H_10_O_6_	92.9308, 150.9950, 285.0392	0.6
Anthocyanin						
39.	1.88	773.2105	Pelargonidin 3-sophoroside-5-glucoside	C_33_H_41_O_20_^+^	611.1784, 517.1150, 269.0663	3.9
40.	3.12	449.1079	Cyanidin-3-*o*-rhamnoside	C_21_H_21_O_10_^+^	302.9065, 287.0544, 259.0607	0.1
41.	5.82	919.2529	Pelargonidin 3-(6″-(E-*p*-coumaroyl) sophoroside-5-glucoside	C_42_H_47_O_22_^+^	757.2007, 431.1008, 269.0447	2.9
42.	6.10	949.2603	Pelargonidin 3-(6″-(E-feruloyl) sophoroside-5-glucoside	C_43_H_49_O_23_^+^	787.2048, 431.0914, 269.0436	0.6
43.	7.05	287.0551	Pelargonidin	C_15_H_11_O_5_^+^	218.9499, 112.9894	0.3
Phenolic and Organic acids						
44.	0.97	115.0029	Maleic acid	C_4_H_4_O_4_	71.0139	2.7
45.	0.98	133.0139	Malic acid	C_4_H_6_O_5_	115.0027, 71.0136	5.6
46.	1.02	191.093	Citric acid	C_6_H_8_O_7_	111.0089, 87.0089, 57.0347	3.6
47.	1.04	175.0241	Ascorbic acid	C_6_H_8_O_6_	130.9661, 87.0090, 59.0125	2.2
48.	1.04	117.0184	Succinic acid	C_4_H_6_O_4_	73.0297	1.4
49.	1.06	103.0030	Malonic acid	C_3_H_4_O_4_	59.0142	4
50.	1.17	89.02350	Lactic acid	C_3_H_6_O_3_	87.0073, 71.0153	2
51.	1.18	193.0344	Glucuronic Acid	C_6_H_10_O_7_	113.0161, 103.0035, 89.0239	0.6
52.	1.18	128.0342	Pyroglutamic Acid	C_5_H_7_NO_3_	128.0360, 82.0295	0.2
53.	1.74	163.0388	*P*-Coumaric acid	C_9_H_8_O_3_	119.0494	1
54.	1.95	223.0607	Sinapic acid	C_11_H_12_O_5_	208.0363, 164.043, 149.0210	2.7
55.	2	197.0431	Syringic acid	C_9_H_10_O_5_	129.0557, 112.988	6.9
56.	2.76	137.0231	Salicylic acid	C_7_H_6_O_3_	93.0351	1.6
57.	2.85	339.0728	Sinapoyl malate	C_15_H_16_O_9_	293.0519, 225.0646, 203.0819	5.1
58.	4.82	147.0441	Cinnamic acid	C_9_H_8_O_2_	103.0542,	0.3
59.	5.21	169.0146	Gallic acid	C_7_H_6_O_5_	169.0146, 65.9993	8.6
60.	7.43	309.0591	Feruloylmalic acid	C_14_H_14_O_8_	104.9520, 193.0182, 294.0264	4.5
Fatty acids and their derivatives						
61.	13.44	295.2272	Dimorphecolic acid	C_18_H_32_O_3_	102.9572, 158.9776, 210.9284	1.5
62.	14.86	271.2281	Juniperic acid	C_16_H_32_O_3_	94.9284, 102.9557, 271.2281	4.9
63.	18.17	277.2160	Linolenic acid	C_18_H_30_O_2_	277.2160	0.7
64.	19.44	227.2004	Myristic acid	C_14_H_28_O_2_	227.2021	0.7
65.	21.57	279.2324	Linoleic acid	C_18_H_32_O_2_	279.2324	1.9
66.	23.47	255.2322	Palmitic acid	C_16_H_32_O_2_	255.2322	1.3
67.	23.64	281.2474	Oleic acid	C_18_H_34_O_2_	281.2474	0.4
Amino acids and their derivatives						
68.	1.04	308.0972	*N*-Fructosyl glutamic acid	C_11_H_19_NO_9_	146.0448	1.3
69.	1.06	146.0447	Glutamic acid	C_5_H_9_NO_4_	128.0349, 102.0557	0.6
70.	1.16	264.1077	*N*-Fructosyl methyl alanine	C_10_H_18_NO_7_	174.0773, 102.0564	0.3
71.	1.18	290.0873	*N*-Fructosyl pyroglutamate	C_11_H_17_NO_8_	200.0561, 128.0349	0.9
72.	1.19	307.1142	*N*-Fructosyl glutamine	C_11_H_20_N_2_O_8_	145.0620, 127.0520	2
73.	1.23	154.0609	Histidine	C_6_H_9_N_3_O_2_	137.0413, 93.0449	1.3
74.	1.23	294.1181	*N*-Fructosyl hydroxy-norvaline	C_11_H_21_NO_8_	132.0306	0.8
75.	1.24	88.0393	Alanine	C_3_H_7_NO_2_	88.0393	0.1
76.	1.25	118.0502	Threonine	C_4_H_9_NO_3_	74.0257	2.8
77.	1.30	116.0710	Valine	C_5_H_11_NO_2_	116.0710	3.4
78.	1.30	292.1394	*N*-Fructosyl isoleucine	C_12_H_23_NO_7_	130.0873	1.1
79.	1.31	278.1237	*N*-Fructosyl Valine	C_11_H_21_NO_7_	116.0724	1
80.	1.39	104.0341	Serine	C_3_H_7_NO_3_	74.0243	1.1
81.	1.54	173.1035	Arginine	C_6_H_14_N_4_O_2_	131.0821	1.1
82.	1.68	180.0655	Tyrosine	C_9_H_11_NO_3_	180.0655, 119.0501, 59.0140	0.1
83.	1.74	262.1289	*N*-pentosyl isoleucine	C_11_H_21_NO_6_	130.0851	1.5
84.	1.8	130.0863	Leucine	C_6_H_13_NO_2_	130.0863, 57.9790	0.3
85.	1.98	164.0707	Phenylalanine	C_9_H_11_NO_2_	147.0459, 103.0541, 72.0087	0.6
86.	2.25	145.0976	Lysine	C_6_H_14_N_2_O_2_	128.0310, 100.9645, 60.9953	3.1
87.	2.57	203.0818	*L*-tryptophan	C_11_H_12_N_2_O_2_	159.0880, 142.0638, 116.0502	1.5
88.	2.64	365.1339	*N*-Fructosyl tryptophan	C_17_H_22_N_2_O_7_	203.0825, 116.0514	1.2
Saccharides						
89.	1.16	503.1608	Raffinose	C_18_H_32_O_16_	503.1608	0.3
90.	1.20	179.0552	Fructose	C_6_H_12_O_6_	89.0245, 71.0140, 59.0138	1
91.	1.35	341.1077	Sucrose	C_12_H_22_O_11_	179.0560, 119.0341, 89.0242	0.4
92.	1.39	181.0705	Mannitol	C_6_H_14_O_6_	112.9852, 71.0134, 59.0138	0.9

(RT: retention time; *m*/*z*: mass-to-charge ratio; PPM: parts per million).

**Table 3:** The effect of the ethanol, omeprazole, low and high doses of *R. sativus* root extract, and low and high doses of synthesized ZnO-NPs on the optical density of Alcian blue’s (pH 2.5) positive secretion within the gastric mucosa.

Ex perimental Groups	Alcian Blue pH (2.5) Optical Density
Group I	10.7 ± 2 *^a^*
Group II	0.2 ± 0.1 *^d^*
Group III	8.5 ± 1.4 *^a^*
Group IV	3.9 ± 0.1 *^c^*
Group V	5.8 ± 0.3 *^b,c^*
Group VI	6.5 ± 0.6 *^b,c^*
Group VII	9.4 ± 1.2 *^a^*

Data are displayed as mean ± SD. ^*a*, *b*, *c*, *d*^ Means with distinct superscripts in a column differ significantly at *p ≤* 0.001.

**Table 4:** Docking results of the glucosinolates, flavonoids, and anthocyanins targeting MMP-10 and ERK.

No.	Compound	ΔG a (kcal/mol)	
		MMP-10	ERK
1	Glucoraphenin	−4.38	−7.83
2	Glucoberteroin	−10.38	−7.41
3	Glucoraphanin	−10.08	−7.51
4	4-Hydroxyglucobrassicin	−10.43	−7.74
5	Gluconapin	−8.40	−6.51
6	Glucoiberverin	−10.13	−7.33
7	Glucobrassicanapin	−10.74	−6.58
8	Glucotropeolin	−10.23	−6.69
9	Glucoerucin	−11.27	−8.10
10	Glucobrassicin	−10.29	−7.77
11	Gluconasturtiin	−9.15	−7.30
12	Neoglucobrassicin	−10.25	−8.14
13	Sulforaphene	−6.86	−5.33
14	Progitrin	−10.67	−7.00
15	Heptyl glucosinolate	−10.64	−8.30
16	Hexyl glucosinolate	−9.10	−7.34
17	Catechin	−4.13	−6.36
18	Methylgalangin	−5.12	−6.74
19	Myricetin	−5.31	−6.26
20	Naringenin	−4.50	−6.48
21	Kaempferol 3-*O*-sophoroside-7-glucoside	−6.99	−9.62
22	Quercetin 3-*p*-coumarylsophoroside-7-glucoside	−8.62	−9.89
23	Kaempferol 3-(2‴-sinapoylsophoroside) 7-glucoside	−7.92	−9.51
24	Kaempferol 3-*O*-feruloyl-sophoroside 7-*O*-glucoside	−7.66	−10.58
25	Dactylin	−6.88	−9.32
26	Rutin	−6.76	−9.45
27	Kaempferol-3-*O*-rhamnoside	−6.10	−8.60
28	Kaempferol 3-*O*-glucosyl-rhamnosyl-glucoside	−6.12	−7.00
29	Nicotiflorin	−6.45	−10.04
30	Narcissin	−7.00	−8.89
31	Kaempferol 3-*O*-xylosyl-rutinoside	−6.24	−10.26
32	Quercetin-3-*O*-glucoside	−4.99	−9.90
33	Kaempferitrin	−7.35	−7.50
34	Isorhamnetin	−5.28	−6.29
35	Astragalin	−5.34	−7.24
36	Isorhamnetin-3-*O*-glucoside	−4.77	−7.85
37	Quercetin	−5.22	−6.33
38	Kaempferol	−5.14	−6.30
39	Pelargonidin 3-sophoroside-5-glucoside	−6.72	−8.47
40	Cyanidin-3-*O*-rhamnoside	−5.55	−7.55
41	Pelargonidin 3-(6″-(E-p-coumaroyl) sophoroside-5-glucoside	−6.69	−10.70
42	Pelargonidin 3-(6″-(E-feruloyl) sophoroside-5-glucoside	−6.66	−10.90
43	Pelargonidin	−4.89	−6.29
44	Reference compound	−7.25	−12.00

## Data Availability

All data generated or analyzed during this study are included in this published article (and its Supplementary Information Files).

## Supplementary Materials

The following supporting information can be downloaded at: https://www.mdpi.com/article/10.3390/life15111710/s1, Figure S1: Base peak chromatogram of *R. sativus* root ethanolic extract; Figure S2: UV spectrum of ZnO nanoparticles of *R. sativus* root extract; Figure S3: Effect of the root extract of *R. sativus* (100 & 200 mg/kg) and the synthesized ZnO-NPs (100 & 200 mg/kg) on the concentration of ulcer severity (a) and ulcer number (b). Group I; (Negative control group). Group II; (Ethanol-induced group). Group III; (Ethanol-Omeprazole 20 mg/kg). Group IV; (Ethanol-root extract 100 mg/kg). Group V; (Ethanol-root extract 200 mg/kg). Group Ⅵ; (Ethanol-ZnO-NPs 100 mg/kg). Group Ⅶ; (Ethanol-ZnO-NPs 200 mg/kg). Results are expressed as mean ± standard error mean (n = 5). Comparisons were made on the basis of the one-way analysis of variance (ANOVA) followed by Tukey’s post hoc test. * (*p* ≤ 0.05), ** (*p* < 0.01), *** (*p* < 0.001), **** (*p* < 0.0001).
