# Supplementary material for: Phyto-Mediated Zinc Oxide Nanoparticles from Raphanus sativus (L.): Metabolomic Insights, Gastroprotective Potential, and Docking-Supported Evidence
Source: Life (Basel). 2025 Nov 5;15(11):1710. doi: 10.3390/life15111710 (PMC12653370; doi:10.3390/life15111710)
Supplement: Supplementary file 1 [file life-15-01710-s001.zip › life-3924245-supplementary.pdf]

Supplementary material

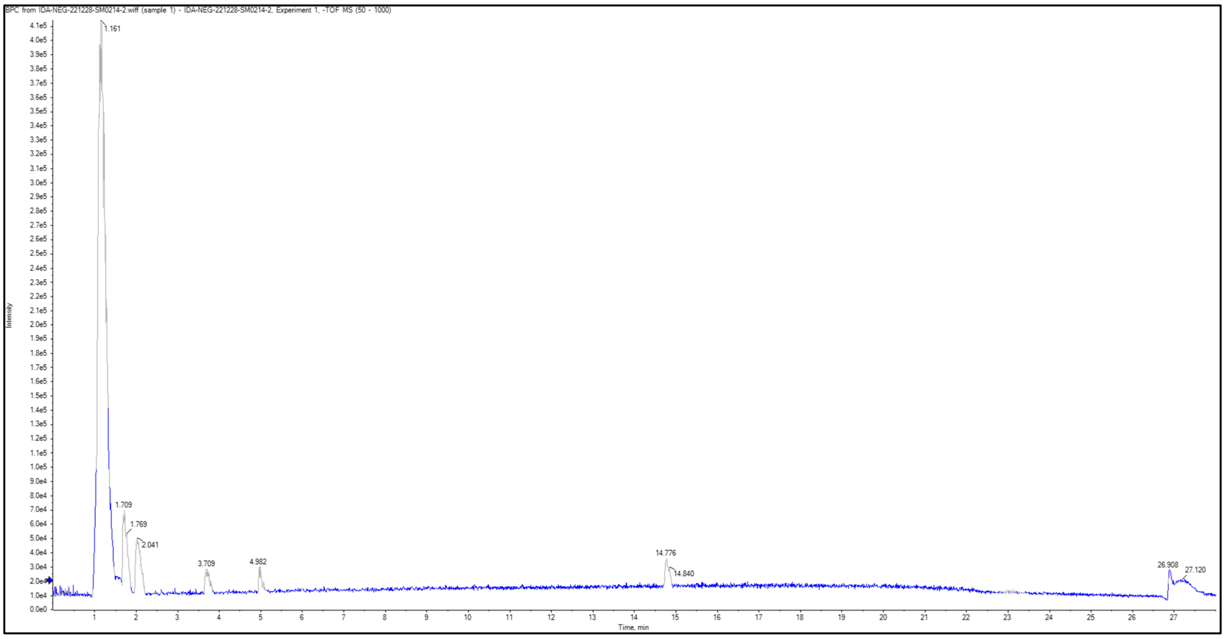

Figure S1. Base peak chromatogram of *R. sativus* root ethanolic extract.

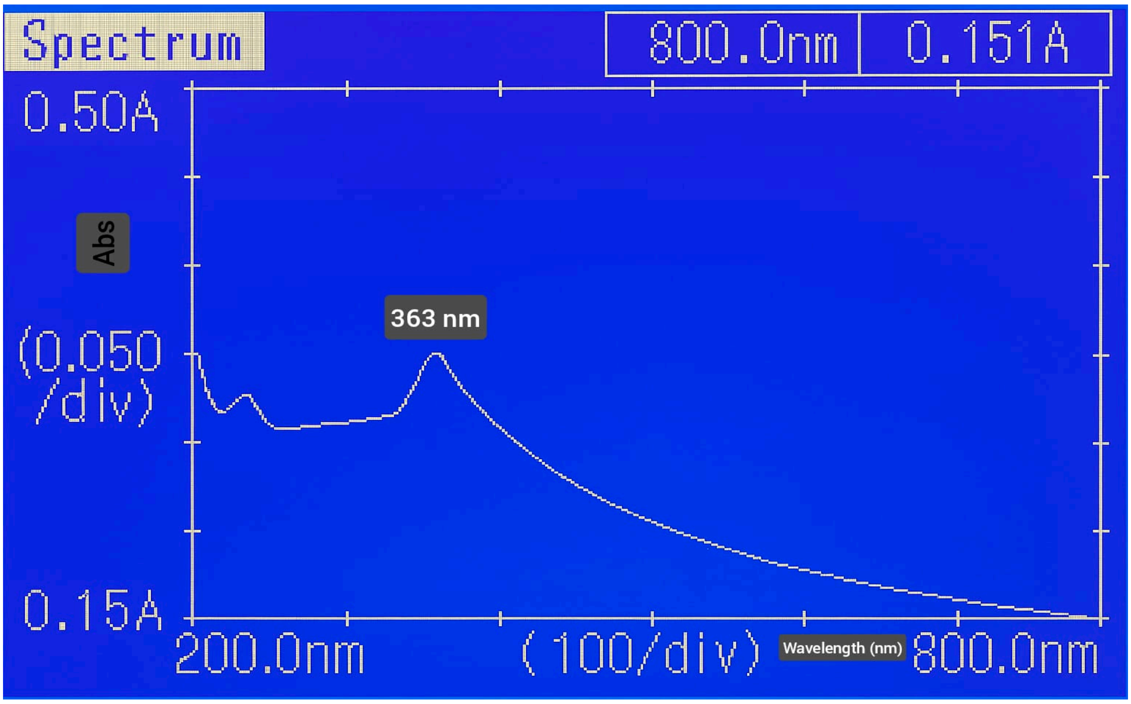

Figure S2. UV spectrum of ZnO nanoparticles of *R. sativus* root extract

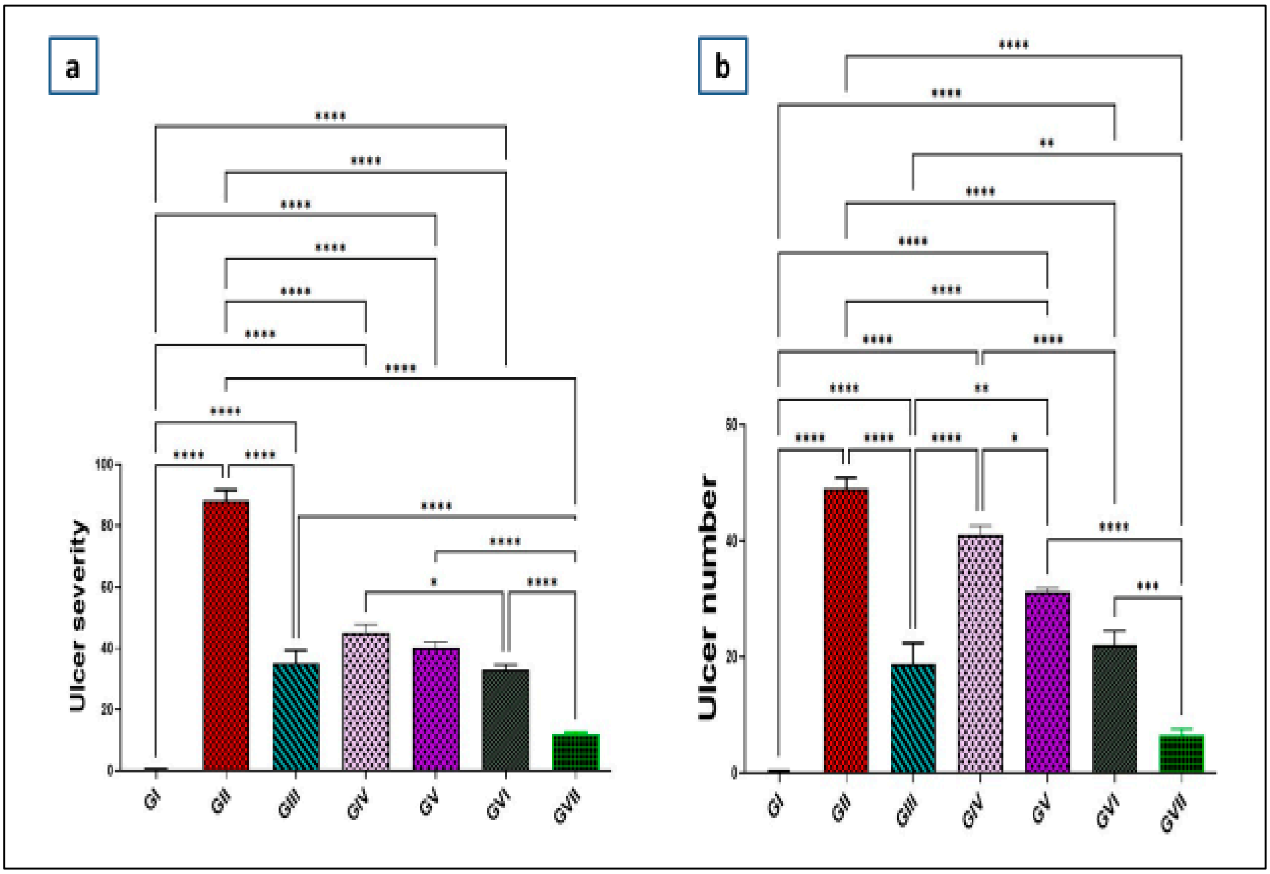

Figure S3. Effect of the root extract of *R. sativus* (100 & 200mg/kg) and the synthesized ZnO-NPs (100&200mg/kg) on the concentration of ulcer severity (a) and ulcer number (b). Group I; (Negative control group). Group II; (Ethanol - induced group). Group III; (Ethanol-Omeprazole 20mg/kg). Group IV; (Ethanol - root extract 100 mg/kg). Group V; (Ethanol-root extract 200 mg/kg). Group VI; (Ethanol-ZnONPs 100 mg/kg). Group VII; (Ethanol-ZnONPs 200 mg/kg). Results are expressed as mean ± standard error mean (n = 5). Comparisons were made on the basis of the one-way analysis of variance (ANOVA) followed by Tukey's post hoc test. * ($P \leq 0.05$) ($P < 0.01$), *** ($P < 0.001$), **** ($P < 0.0001$).**
